# Mapping male circumcision for HIV prevention efforts in sub-Saharan Africa

**DOI:** 10.1186/s12916-020-01635-5

**Published:** 2020-07-07

**Authors:** Michael A. Cork, Kate F. Wilson, Samantha Perkins, Michael L. Collison, Aniruddha Deshpande, Jeffrey W. Eaton, Lucas Earl, Emily Haeuser, Jessica E. Justman, Damaris K. Kinyoki, Benjamin K. Mayala, Jonathan F. Mosser, Christopher J. L. Murray, John N. Nkengasong, Peter Piot, Benn Sartorius, Lauren E. Schaeffer, Audrey L. Serfes, Amber Sligar, Krista M. Steuben, Frank C. Tanser, John D. VanderHeide, Mingyou Yang, Njeri Wabiri, Simon I. Hay, Laura Dwyer-Lindgren

**Affiliations:** 1 Institute for Health Metrics and Evaluation, University of Washington, Seattle, WA USA; 2 Department of Infectious Disease Epidemiology, Imperial College London, London, UK; 3 ICAP, Mailman School of Public Health, Columbia University, New York, NY USA; 4 Vagelos College of Physicians and Surgeons, Columbia University, New York, NY USA; 5 DHS program, ICF International, Rockville, MD USA; 6 Department of Health Metrics Sciences, School of Medicine, University of Washington, Seattle, WA USA; 7 Africa Centres for Disease Control and Prevention, African Union, Addis Ababa, Ethiopia; 8 London School of Hygiene & Tropical Medicine, London, UK; 9 School of Nursing and Public Health, University of KwaZulu-Natal, Durban, South Africa; 10 Africa Health Research Institute, KwaZulu-Natal, South Africa; 11 Centre for the AIDS Programme of Research in South Africa (CAPRISA), University of KwaZulu-Natal, Durban, South Africa; 12 Research Department of Infection & Population Health, University College London, London, UK; 13 HIV/AIDS, STIs & TB Research Programme, Human Sciences Research Council, Pretoria, South Africa

**Keywords:** Male circumcision, Medical male circumcision, Voluntary medical male circumcision, HIV, HIV prevention, Intervention, Mapping, Africa, Geospatial, Geostatistics, Spatial statistics

## Abstract

**Background:**

HIV remains the largest cause of disease burden among men and women of reproductive age in sub-Saharan Africa. Voluntary medical male circumcision (VMMC) reduces the risk of female-to-male transmission of HIV by 50–60%. The World Health Organization (WHO) and Joint United Nations Programme on HIV/AIDS (UNAIDS) identified 14 priority countries for VMMC campaigns and set a coverage goal of 80% for men ages 15–49. From 2008 to 2017, over 18 million VMMCs were reported in priority countries. Nonetheless, relatively little is known about local variation in male circumcision (MC) prevalence.

**Methods:**

We analyzed geo-located MC prevalence data from 109 household surveys using a Bayesian geostatistical modeling framework to estimate adult MC prevalence and the number of circumcised and uncircumcised men aged 15–49 in 38 countries in sub-Saharan Africa at a 5 × 5-km resolution and among first administrative level (typically provinces or states) and second administrative level (typically districts or counties) units.

**Results:**

We found striking within-country and between-country variation in MC prevalence; most (12 of 14) priority countries had more than a twofold difference between their first administrative level units with the highest and lowest estimated prevalence in 2017. Although estimated national MC prevalence increased in all priority countries with the onset of VMMC campaigns, seven priority countries contained both subnational areas where estimated MC prevalence increased and areas where estimated MC prevalence decreased after the initiation of VMMC campaigns. In 2017, only three priority countries (Ethiopia, Kenya, and Tanzania) were likely to have reached the MC coverage target of 80% at the national level, and no priority country was likely to have reached this goal in all subnational areas.

**Conclusions:**

Despite MC prevalence increases in all priority countries since the onset of VMMC campaigns in 2008, MC prevalence remains below the 80% coverage target in most subnational areas and is highly variable. These mapped results provide an actionable tool for understanding local needs and informing VMMC interventions for maximum impact in the continued effort towards ending the HIV epidemic in sub-Saharan Africa.

## Background

Despite considerable progress made in combating the HIV epidemic in the past three decades, HIV/AIDS remains the single largest cause of health loss among men and women of reproductive age in sub-Saharan Africa [1]. In 2017, an estimated 25.9 million people were living with HIV in sub-Saharan Africa, with over one million newly infected in that year [1]. Voluntary medical male circumcision (VMMC), defined as the complete surgical removal of the foreskin [2], has emerged in recent years as an effective intervention to reduce HIV transmission risk. Typically limited to boys and men ages 10 years and older, VMMC reduces the risk of female-to-male transmission of HIV by 50–60% [3–5]. Outside of a clinical trial setting, several long-term assessments have confirmed VMMC’s protective role in reducing HIV burden [6, 7], and even demonstrated a rise in effectiveness as high as 73% [6]. Recent evidence justifies the VMMC implementation now in progress [8–11], bolstered by additional meta-analyses [10, 12]. Moreover, VMMC is one-time, efficient, safe, cost-effective, and the only HIV prevention method specifically aimed at heterosexual men—a group with historically low HIV testing rates [13]. VMMC provides a unique opportunity to increase awareness of HIV status among millions of men and boys who might otherwise forgo HIV testing [14]. Protective effects also extend to partners of circumcised men; female partners have decreased risks of genital ulcer disease, cervical cancer, trichomonas infection, and bacterial vaginosis, among other outcomes [15]. Further, there is growing evidence that VMMC reduces risk of HIV transmission among men who have sex with men (MSM) [16].

As a result of this compelling evidence, the regional scale-up of VMMC has been immense. The World Health Organization (WHO) and the Joint United Nations Programme on HIV/AIDS (UNAIDS) designated 14 priority countries in southern and eastern Africa with high national HIV prevalence and low coverage of male circumcision (MC), as of 2017 consisting of Botswana, Eswatini, Ethiopia, Kenya, Lesotho, Malawi, Mozambique, Namibia, Rwanda, South Africa, Tanzania, Uganda, Zambia, and Zimbabwe [17] (in 2018 South Sudan was included, bringing the total priority countries to 15) [18]. Between 2008 and 2017, nearly 18.6 million VMMCs were reported in priority countries with the support of national and global programs including the US President’s Emergency Plan for AIDS Relief (PEPFAR) and the Global Fund to Fight AIDS, Tuberculosis and Malaria [2, 13]. The WHO estimated that VMMC campaigns had already averted 230,000 new HIV infections by 2017, with over one million infections anticipated to be averted by 2030 [2]. An ambitious target was set in 2012 to reach 80% MC coverage of men ages 15–49 years by 2015, and while most countries are still working towards meeting this coverage, new complementary targets focus on 90% coverage in younger males ages 10–29 [19]. The WHO maintains a target of 25 million total circumcisions by 2020 in priority countries to help achieve these high rates of MC [20].

Notwithstanding this unprecedented scale-up of VMMC, there are currently no comprehensive estimates of MC prevalence in sub-Saharan Africa that report over multiple years and at a subnational level. UNAIDS presents national-level estimates compiled from population-based surveys in their annual global updates, but these are limited to the select countries and years in which surveys were administered [20]. While there is evidence of substantial subnational variation of MC coverage [21–24], past studies that estimate MC prevalence in multiple countries in Africa often report only at the national level [25], and those that do report subnational MC prevalence include only a subset of available survey data and are confined to individual countries and few years [21, 22]. In the majority of existing estimates and in our current analysis, MC prevalence encompasses not only MC performed by VMMC, but also traditional circumcision, which is influenced by values and beliefs that differ across cultural, religious, and ethnic identities [24, 26, 27]. While traditional circumcision is often non-medical, taking place in a non-clinical setting by a traditional provider with no formal medical training [27], it can also include medical MC carried out in a clinical setting by a trained and supervised service provider [28]. Traditional circumcisions that occur in a medical setting may have a similar procedure to that of VMMC, but in our analysis, VMMC refers only to the intervention strategy adopted in 2008. Due to this variation in practice, evidence that traditional circumcision is protective against HIV is mixed [18, 29, 30]. Nonetheless, yearly estimates of MC prevalence can provide valuable information on the unmet needs of men amenable to VMMC who have not undergone any form of MC. Further, tracking MC prevalence over multiple years provides insight into how quickly VMMC efforts are raising MC coverage, and allows for additional precision in MC estimates by leveraging strength across different survey years and in neighboring countries.

The absence of comprehensive subnational MC estimates is troubling given the demonstrated acute subnational variation in HIV prevalence across sub-Saharan Africa [31] and the growing evidence that MC prevalence may play a role in driving the spatial distribution of the HIV epidemic [21]. Various studies have emphasized that HIV prevention services that focus on the local epidemiological context are able to achieve greater impact than uniformly distributed services [32–35], but the lack of robust subnational information on MC coverage precludes many countries from adopting this approach [36]. To track VMMC scale-up and progress towards coverage targets, the WHO reports the number of VMMCs performed by country and year [2]. VMMC monitoring and report systems are often reported in parallel to or embedded within national health information systems and data processing often involves multiple partners, which can present a challenge to interpretation of absolute numbers given it can increase the likelihood of errors, especially as it relates to MC coverage changes at a subnational level [37]. In addition, the absolute number of newly circumcised men does not translate to MC prevalence in a specified age group, making it difficult to assess progress towards coverage goals. To best inform HIV prevention efforts and describe progress towards coverage targets, local MC coverage estimates should be produced based on data sources independent of reported VMMCs, should cover all locations, should support tracking changes in MC coverage over time, and should capture patterns of the number of circumcised and uncircumcised men over time and space.

In this analysis, we provide the first comprehensive, annual estimates of MC coverage and numbers of circumcised and uncircumcised men ages 15–49 residing in 38 countries in sub-Saharan Africa at local (5 × 5 km) and subnational (first and second administrative levels) spatial resolutions from 2000 to 2017. We do not restrict our study to the 14 VMMC priority countries given the availability of data in 38 countries in sub-Saharan Africa and the shortage of current estimates of MC coverage across the continent. Further, there is evidence that some countries or subnational areas may later become relevant to policymakers; for example, in 2018, South Sudan initiated a pilot VMMC program [18]. Nonetheless, we focus the majority of our analysis on the priority countries selected for VMMC campaigns because of their present relevance to decision-makers and stakeholders in allocating resources to VMMC services. In these countries, we examine the local distribution of MC prevalence and uncircumcised men in 2017 and describe changes in MC prevalence and the number of circumcised men at subnational levels in relation to the onset of VMMC campaigns. Finally, we assess countries’ progress towards the 80% MC coverage target at national and subnational levels.

## Methods

### Input data

We compiled a dataset of 38,747 geo-referenced observations from 109 household surveys conducted between 2000 and 2017 in 38 countries in sub-Saharan Africa as defined by the Global Burden of Disease (GBD) study [11]. We assembled this dataset through a review of major survey series (Demographic and Health Survey [DHS], AIDS Indicator Survey [AIS], Multiple Indicator Cluster Survey [MICS], Core Welfare Indicators Questionnaire Survey [CWIQ], Population-based HIV Impact Assessment Survey [PHIA]) and surveys tagged with the “circumcision” keyword in the Global Health Data Exchange [38]. We included all surveys that asked men about their circumcision status, were sampled from the general population, and contained subnational geographical information (Supplementary Table 1 in Additional file 1). We excluded countries in sub-Saharan Africa with no data availability from our analysis. We also excluded the island nation of São Tomé and Príncipe, despite having one data source with low sample size, due to evidence that its MC coverage patterns were substantially lower than the regional trends, which would break our implicit model assumption that areas that are close in space and time borrow information on MC prevalence (Supplementary Table 2). Survey data were not required to distinguish between medical and traditional male circumcision, and in the few surveys that included both, MC prevalence was derived from summing across both variables. Survey data were originally in two forms: survey microdata (i.e., individual-level survey responses) or survey reports (Supplementary Table 1). For surveys with available microdata, we extracted variables related to age, MC status, location, and survey weights. After subsetting the data to ages 15–49 years and excluding observations with missing information on any of these variables (4.0% of all observations), we aggregated the data by calculating the survey-weighted MC prevalence at the most precise spatial resolution available. The ideal spatial resolution was a latitude and longitude point that represented the location of the survey cluster (point-level data), but when not available, we geo-located survey microdata to the smallest geographical areal unit (termed a polygon), typically representing an administrative unit. In instances where data were matched to a polygon rather than specific GPS coordinates, these data were “resampled” proportional to the underlying population in the areal unit to mimic point data. The population data used for this analysis were obtained from WorldPop [39]. The methods for sampling point locations from areas associated with areal data are consistent with previous studies using model-based geostatistics [31, 40–43]. Weighting by sample size, 70.7% of all data were associated with GPS coordinates, while the remaining data were associated with polygons and resampled using this approach.

Several survey reports presented MC estimates for age groups other than 15–49 years (five sources representing 1.4% of the total effective sample size; Supplementary Table 3 in Additional file 1). In order to incorporate data from these reports, we used a cross-walking model—an approach for linking disparate data sources (in this case data sources reporting for different age groups)—that used existing microdata and a linear regression to convert the prevalence in the reported age range to the standard 15–49 age range. The age cross-walk is consistent with the method previously used in the geospatial modeling of HIV [31]; this process calculated a new sample size that reflects our confidence in the estimate of MC prevalence as a function of the uncertainty in our linear model and the original sample size. The distribution of geo-located MC prevalence data is shown in Supplementary Fig. 1, and a list of sources is also located in Supplementary Table 1 in Additional file 1. We excluded three data sources in Nigeria because lower coverage contradicted several independent DHS surveys in the same country, and there was evidence that at least one of the reports had been previously flagged for data validation issues. The excluded data and the reasoning for their exclusion can be found in Supplementary Table 2. Additional methodological details on data identification and processing are also presented in section 2 in Additional file 1.

### Data analysis

We modeled MC prevalence using a Bayesian spatially and temporally explicit generalized linear mixed effects model developed previously for an analysis of HIV prevalence and updated for this analysis (Supplementary Fig. 2 in Additional file 1) [31]. Due to computational constraints and to allow for regional differences in the temporal and spatial autocorrelation in MC prevalence, we fit separate models for four geographically contiguous regions in sub-Saharan Africa adapted from the Global Burden of Diseases, Injuries, and Risk Factors study (Supplementary Fig. 3 in Additional file 1) [1]. We excluded countries in sub-Saharan Africa with no data availability, with the exception of São Tomé and Príncipe, which was excluded despite having one data source due to evidence that its MC patterns were substantially different from the regional trends. Within our Bayesian geostatistical model, MC coverage was modeled as binomial count data with a logit link function and the linear model included an intercept, a fixed effect on year, a country-level random effect, an autocorrelated spatiotemporal random effect, and an uncorrelated error term (nugget effect). Unlike similar past analyses [40–43], we did not use covariates for this analysis given that predictive covariates such as ethnicity, culture, or religion were not available at our model’s spatial and temporal resolution. Due to the absence of viable covariates and the limited availability of survey data in the most recent year, extrapolation of our estimates to later years in the study period was often informed by the regional time trend provided by the fixed effect on year. After fitting the model, we generated 1000 draws of MC prevalence from the approximated joint posterior distribution at each 5 × 5-km grid cell across each model region and year in the study period. We calculated point estimates for each grid cell as the mean of these draws, and we designated the 95% uncertainty intervals as the 2.5th and 97.5th percentiles of these draws.

In addition to estimates of MC prevalence on a 5 × 5-km spatial grid, we produced estimates of MC prevalence for first and second administrative level units. We constructed these estimates by calculating population-weighted averages of MC prevalence for each grid cell or fraction of a grid cell within a given first or second administrative level unit as defined by the Database of Global Administrative Areas (GADM) [44]. We list the names and the number of designated first and second administrative level units in each country in Supplementary Table 4 in Additional file 1. Furthermore, we derived estimates of the number of circumcised and uncircumcised men in each administrative unit by multiplying estimated MC prevalence or uncircumcised prevalence, respectively, in each grid cell by the corresponding WorldPop population estimates of men ages 15–49 and aggregating to administrative units. Finally, we calculated the posterior probability of exceeding 80% MC prevalence in 2017 at the 5 × 5-km level and among administrative units as the percentage of draws from the approximated posterior distribution in which MC prevalence was 80% or greater. Throughout our analysis, we qualify statements as statistically significant if the posterior probability of that statement exceeds 95%. All models were fitted using an integrated nested Laplace approximation in the R-INLA package, version 3.3.2 [45, 46].

We performed both in-sample and out-of-sample model validation using a spatiotemporal fivefold cross-validation strategy. Out-of-sample validation metrics for MC coverage indicate good model fit with spatial stratification at the second administrative level (Supplementary Table 5 in Additional file 1), including mean error (0.6 percentage-points), root-mean-square error (8.7 percentage-points), and 95% coverage of predictive intervals (97.0%). Additional results and details on data preparation, modeling, estimation, and validation can be found in Additional file 1.

## Results

### Regional patterns of MC prevalence

**Fig. 1:**
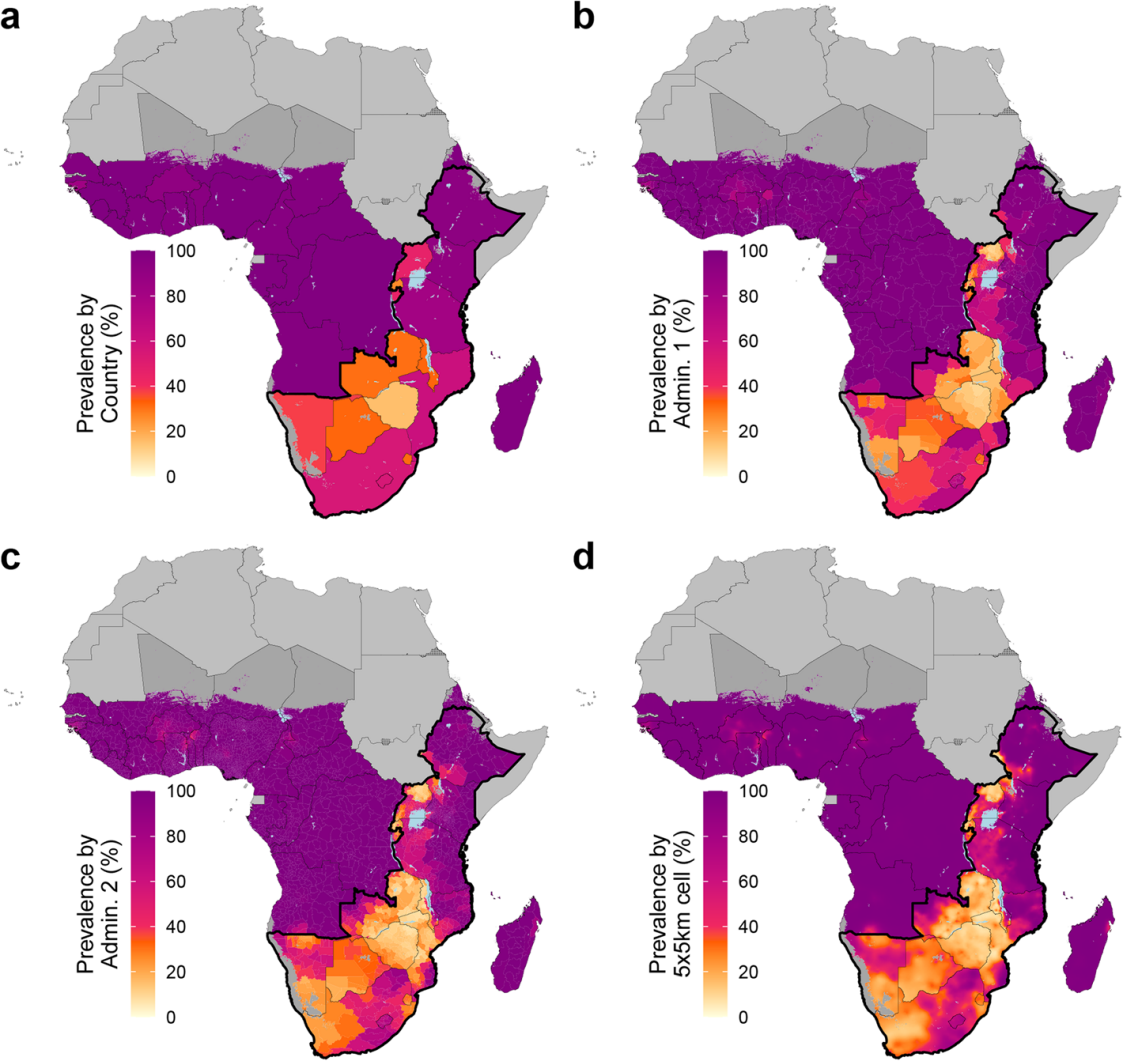
Estimated male circumcision prevalence, adult men ages 15–49 years, 2017. Male circumcision prevalence among adult men ages 15–49 years in 2017 at the country level (**a**), first administrative unit level (**b**), second administrative unit level (**c**), and 5 × 5-km grid-cell level (**d**). Maps reflect administrative boundaries, land cover, lakes, and population. Grid cells with fewer than ten people per 1 × 1-km [39] and classified as “barren or sparsely vegetated” [47] are colored in dark gray. Countries in light gray were not included in the analysis. Outlined by a thick black border are priority countries for VMMC campaigns in southern and eastern Africa, as identified by the WHO and UNAIDS

**Fig. 2:**
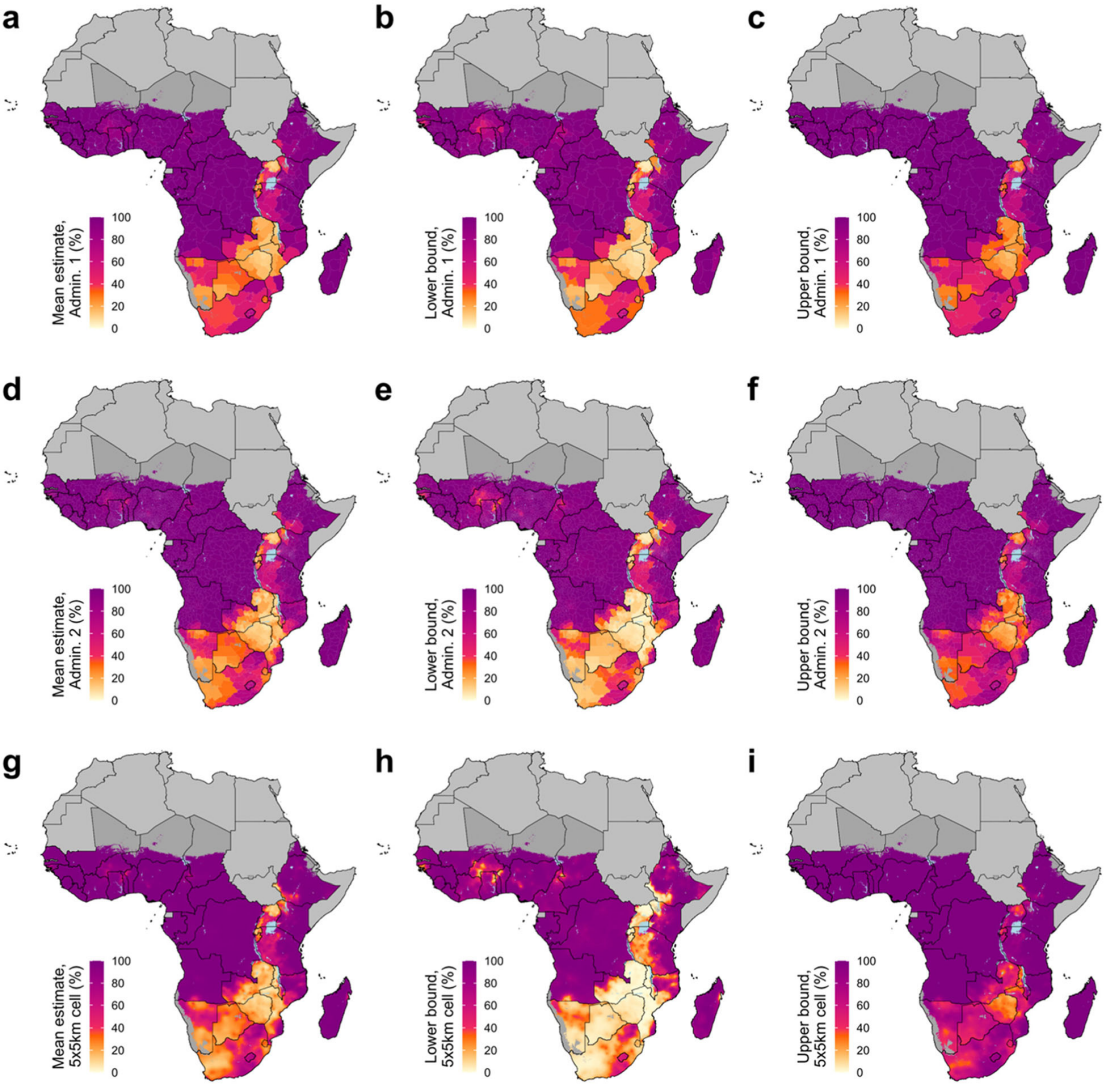
Mean, lower, and upper bounds of estimated male circumcision, ages 15–49, 2017. MC prevalence among males ages 15–49 in 2017 at the first administrative unit level (**a–c**), second administrative unit level (**d–f**), and 5 × 5-km grid-cell level (**g–i**). Mean estimates and lower and upper bounds of the 95% uncertainty intervals are shown in the left, middle, and right columns, respectively. Maps reflect administrative boundaries, land cover, lakes, and population. Grid cells with fewer than ten people per 1 × 1-km [39] and classified as “barren or sparsely vegetated” [47] are colored in dark gray. Countries in light gray were not included in the analysis

Across sub-Saharan Africa, there were stark regional contrasts in estimated MC prevalence, with high levels of MC prevalence in western and central sub-Saharan Africa, low levels of MC prevalence in southern sub-Saharan Africa, and variable levels of MC prevalence in eastern sub-Saharan Africa (Figs. 1 and 2, Supplementary Figs. 4–6). Estimated MC prevalence in most countries in western and central sub-Saharan Africa was largely uniformly high; estimated coverage was greater than 85% in > 95% of second administrative level units in these countries from 2000 to 2017. Conversely, estimated MC prevalence in southern and eastern sub-Saharan African countries with lower levels of MC overall was heterogeneous in space and time. The 14 priority countries identified by WHO/UNAIDS for VMMC were among the group with lower coverage: 13 of these countries (excluding Ethiopia) were among the 15 countries with the lowest estimated national MC prevalence at the start of VMMC campaigns in 2008 (along with Burundi and Guinea-Bissau).

### Geographic variations of MC prevalence and the number of uncircumcised men in 2017

**Fig. 3:**
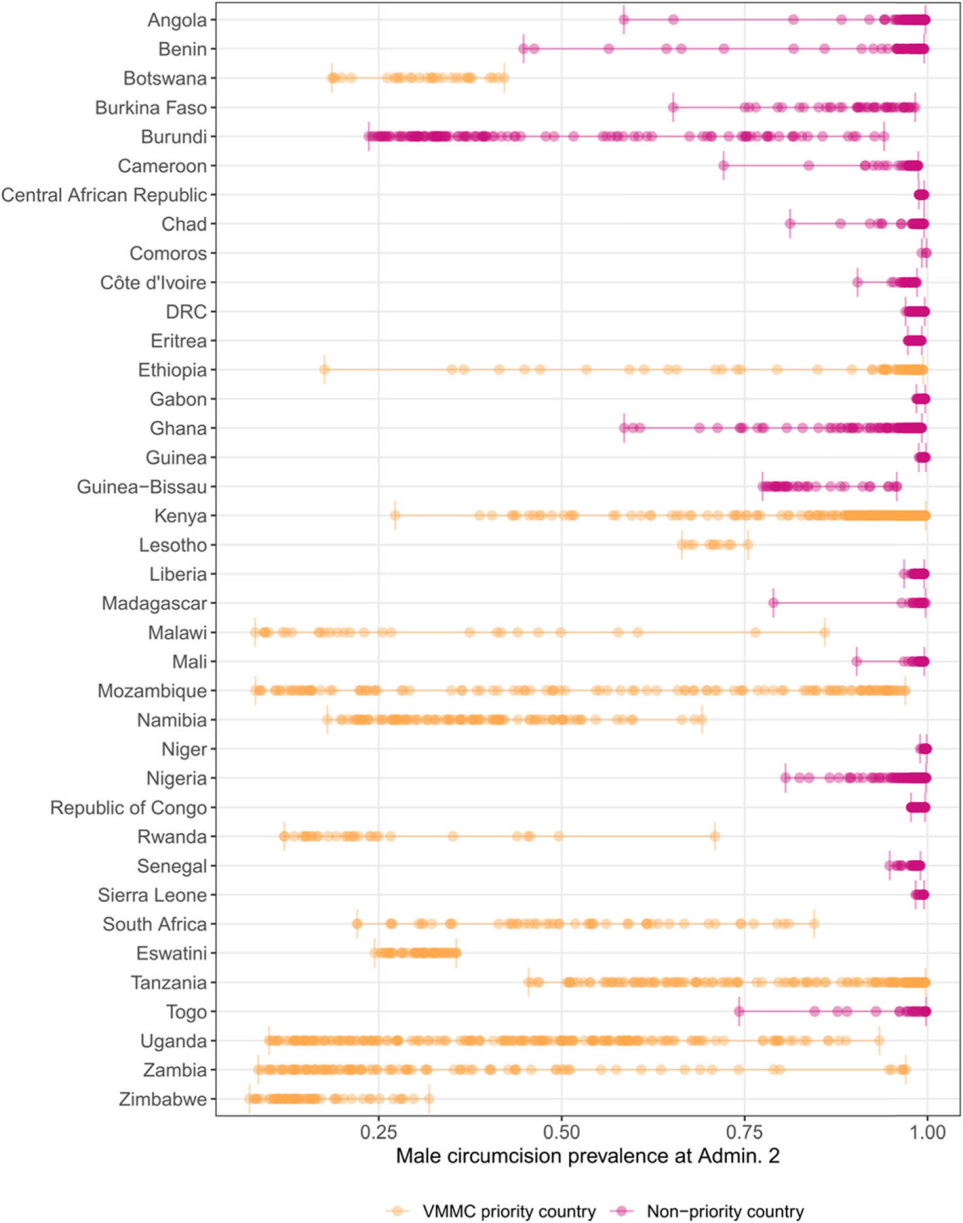
Range of estimated male circumcision prevalence at the second administrative level, 2017. Estimated range of male circumcision prevalence for each second administrative level unit in 2017 by country. Each point represents the estimated prevalence of a single second administrative level unit within a country in 2017, and the vertical bars indicate the prevalence of the highest and lowest second administrative level units in 2017 by country. Color indicates whether or not the country was a priority country for VMMC campaigns, as identified by the WHO and UNAIDS

Within priority countries, estimated MC prevalence varied substantially among first and second administrative level units. In Kenya, with a high estimated national prevalence of 90.8% (95% uncertainty interval, 86.8–93.8%), MC prevalence varied at the first administrative level from 99.6% (98.8–99.9%) in the Makueni county to 40.9% (24.4–57.8%) in the Turkana county in 2017. In Uganda, with a more moderate national prevalence of 45.6% (38.6–52.1%), MC prevalence varied at the first administrative level from 87.4% (79.2–93.5%) in the Kasese district to 10.0% (4.4–18.4%) in the Pader district in 2017. These considerable subnational differences in prevalence were less apparent in low-prevalence settings, but still notable; for example, in Zimbabwe (national prevalence, 15.3% [11.8–19.3%]), MC prevalence varied at the first administrative level from 28.3% (16.6–41.4%) in the Bulawayo province to 9.8% (6.1–14.5%) in the Mashonaland Central province in 2017. There was a more than twofold difference in MC prevalence between the first administrative level units with the highest and lowest estimated prevalence in most (12 of 14; barring Lesotho and Eswatini) priority countries in 2017. This heterogeneity in MC prevalence increased at the second administrative level, where nearly half (6 of 14) of the VMMC priority countries had more than a fivefold difference between their second administrative level units with the lowest and highest estimated prevalence in 2017 (Fig. 3).

**Fig. 4:**
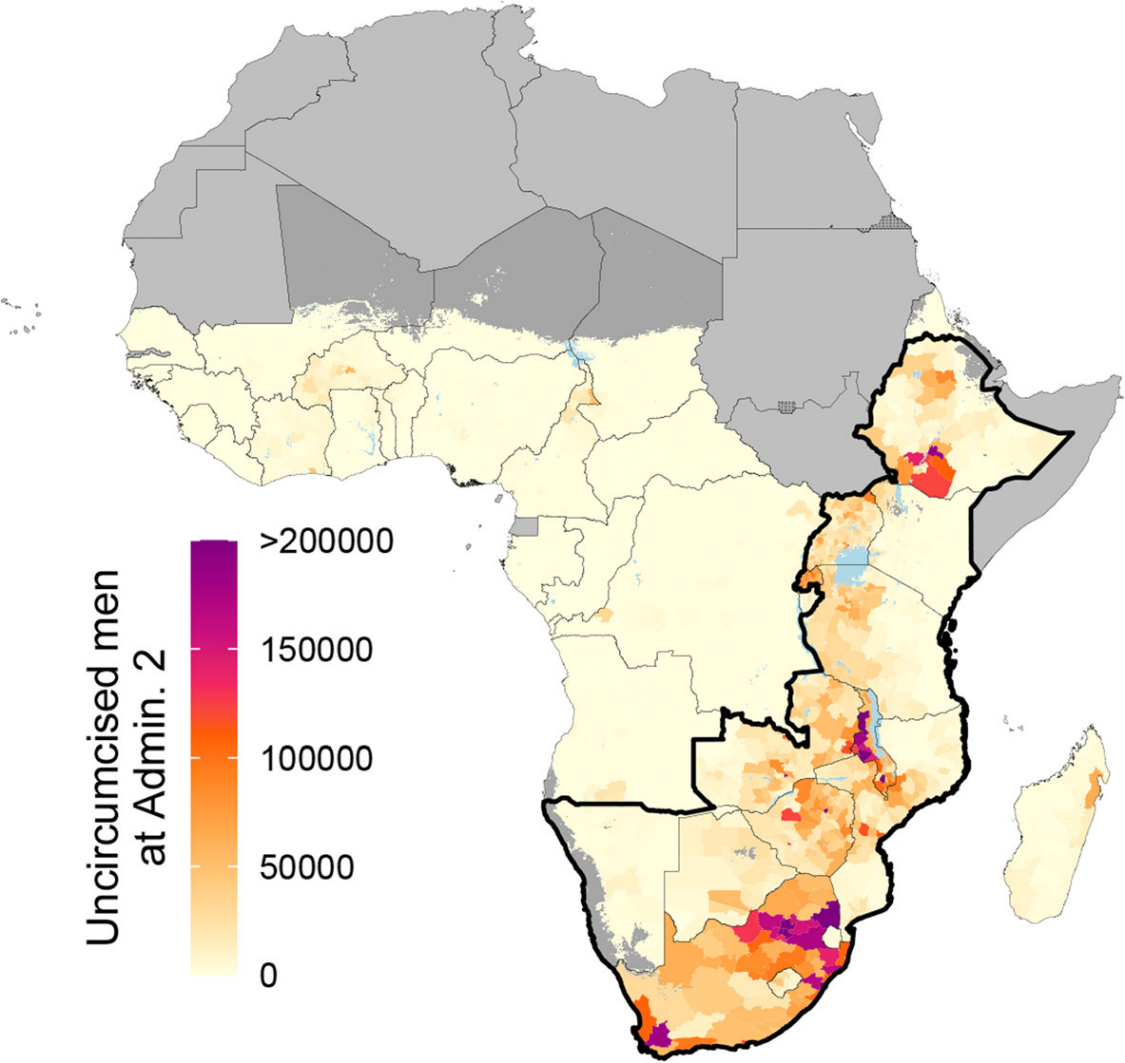
Estimated number of uncircumcised adult men at the second administrative level, 2017. Number of uncircumcised men ages 15–49 years in 2017 at the second administrative unit level. Maps reflect administrative boundaries, land cover, lakes, and population. Grid cells with fewer than ten people per 1 × 1-km [39] and classified as “barren or sparsely vegetated” [47] are colored in dark gray. Countries in light gray were not included in the analysis. Outlined by a thick black border are priority countries for VMMC campaigns in southern and eastern Africa, as identified by the WHO and UNAIDS

We estimated that a large proportion of uncircumcised men were concentrated in a small number of geographical areas. At the country level in 2017, roughly half (49.8%) of the estimated 34.5 (32.9–36.1) million uncircumcised men in sub-Saharan Africa resided in South Africa, Uganda, Zambia, and Zimbabwe, compared to 13.6% of all men in sub-Saharan Africa aged 15–49 residing in those countries. The majority (30.5 [29.2–32.0] million; 88.5% [86.2–90.3%]) of uncircumcised men in sub-Saharan Africa were concentrated in the 14 priority countries. Furthermore, within priority countries in 2017, more than half (50.3%) of all uncircumcised men were concentrated in just 123 (9.1%) second administrative level units, compared to 29.2% of all men aged 15–49 in priority countries residing in those administrative units (Fig. 4). We also observed this heterogeneous spatial distribution of uncircumcised men within each priority country. In 12 of 14 countries (excluding Eswatini and Rwanda), more than half of the country’s estimated uncircumcised men resided in only 30% or fewer second administrative level units. In eight countries, more than half of the country’s uncircumcised men lived in 20% or fewer second administrative level units.

### Time trends in MC prevalence and the number of circumcised men

**Fig. 5:**
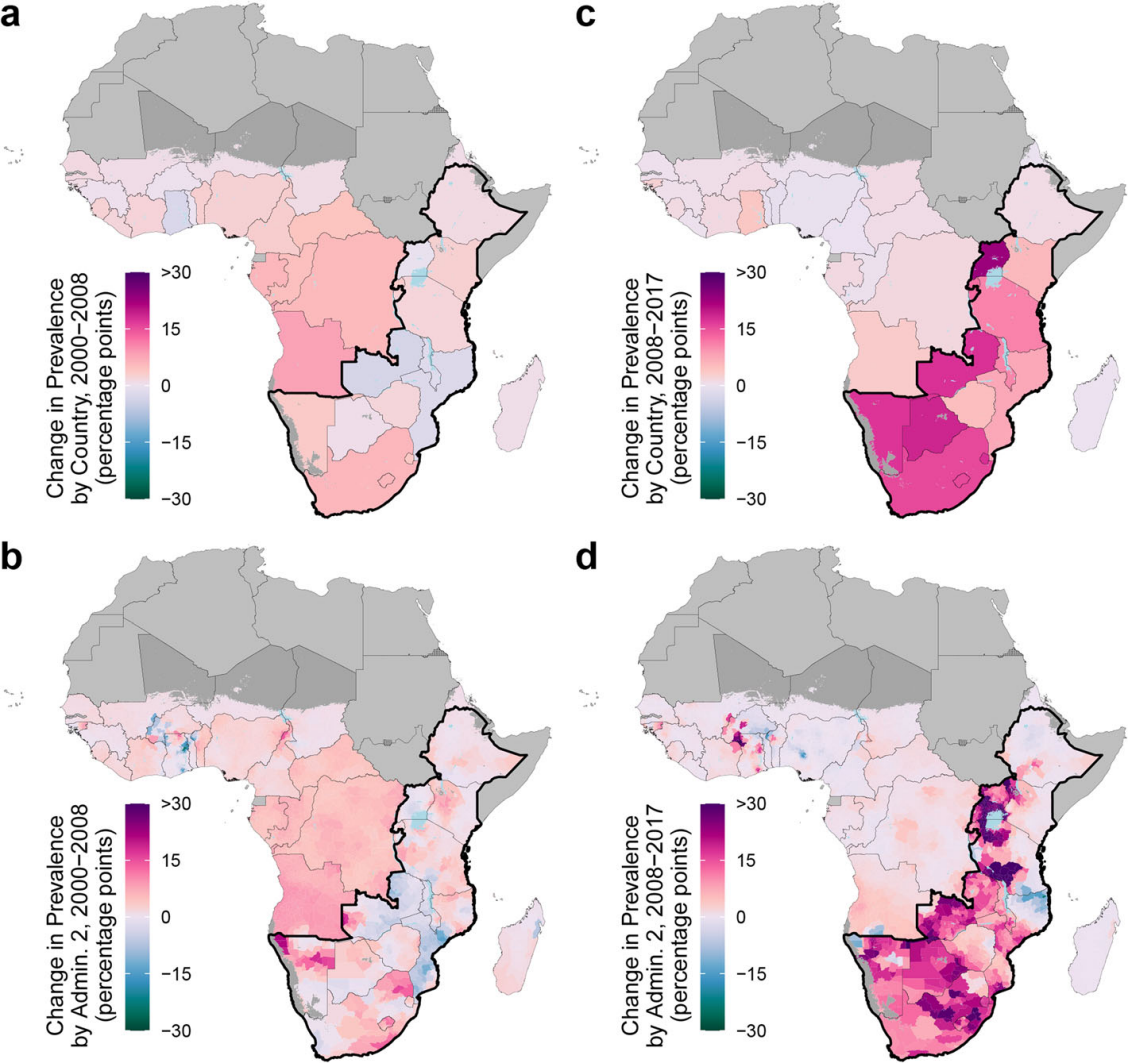
Estimated change in male circumcision prevalence, adult men age 15–49, in 14 priority countries. Absolute change at the country level in male circumcision prevalence among adults age 15–49 between 2000 and 2008 (**a**) and between 2008 and 2017 (**b**). Absolute change at the second administrative unit level in male circumcision prevalence between 2000 and 2008 (**c**) and between 2008 and 2017 (**d**). Maps reflect administrative boundaries, land cover, lakes, and population. Grid cells with fewer than ten people per 1 × 1-km [39] and classified as “barren or sparsely vegetated” [47] are colored in dark gray. Countries in light gray were not included in the analysis. Outlined by a thick black border are priority countries for VMMC campaigns in southern and eastern Africa, as identified by the WHO and UNAIDS

Estimated MC prevalence changed considerably over time, and the temporal trends were marked by the onset of large-scale VMMC campaigns in priority countries from 2008 onwards (Fig. 5, Supplementary Fig. 7). From 2000 to 2008, prior to the VMMC campaigns’ inception, the estimated mean national MC prevalence stagnated in all priority countries; the model does not predict a statistically significant (posterior probability > 95%) increase or decrease in any country’s national MC prevalence over this period. At subnational levels over the same time period, we estimated an increase in MC prevalence in 60.1% (37.1–80.1%) and 58.7% (43.7–71.6%) of first and second administrative level units, respectively. However, among those administrative units with an estimated increase in MC prevalence, the median estimated increase was slight—2.0 and 2.2 percentage-points in first and second administrative level units, respectively. Furthermore, only 0.7% of our estimated increases at all first administrative level units were statistically significant (posterior probability > 95%), indicating high uncertainty in MC temporal trends from 2000 to 2008.

In contrast, and corresponding with the onset of the VMMC campaigns, the estimated number of circumcised men and MC coverage increased in priority countries from 2008 to 2017. The total number of circumcised men in priority countries rose by 21.5 (19.9–23.0) million in this period, from 42.5 (41.8–43.2) million in 2008 to 64.0 (62.5–65.3) million in 2017. Over this same period, national MC coverage increased in all priority countries, and this increase was statistically significant (posterior probability > 95%) in all countries barring Ethiopia. Within priority countries, however, the magnitude of increase varied; for example, estimated MC prevalence rose by 1.0 (− 1.7, 3.7) percentage-points in Ethiopia and 22.9 (15.2–31.3) percentage-points in Lesotho. At subnational levels, we estimated an increase in MC prevalence in 91.9% (86.0–95.8%) and 84.7% (78.1–90.0%) of all first and second administrative level units, respectively, in priority countries. Among those administrative units with an estimated increase in MC prevalence, the median estimated increase was 15.0 percentage-points and 12.2 percentage-points in first and second administrative level units, respectively. These estimated increases were also more certain; 65.1% of the estimated increases in first administrative level units were statistically significant (posterior probability > 95%).

While there was a generalized surge in estimated MC prevalence that coincided with the onset of VMMC efforts from 2008 onwards, these broad trends mask large subnational disparities. Three of the 14 priority countries—Tanzania, Mozambique, and Ethiopia—contained areas where estimated MC prevalence increased as well as areas where estimated MC prevalence decreased among first administrative level units; this was true for seven priority countries at the second administrative level. While we estimated a decrease in 121 (8.9%) of second administrative level units in priority countries after the onset of VMMC campaigns in 2008, the magnitude of these declines was small (mean across units, 2.6 [0.4–4.9] percentage-points) and the impacted areas generally had high coverage in 2008 (mean across units, 90.5% [89.3–91.6]) and therefore still had high coverage in 2017 (mean across units, 88.0% [85.8–89.9]). In certain areas, these differences were substantial. For example, in Mozambique, where the national estimated MC prevalence rose by 7.6 (1.4–13.3) percentage-points from 2008 to 2017, the difference in coverage at the second administrative level ranged from an estimated  -23.0 (-45.2,  -3.5) percentage-point change in the Muidumbe district (from 88.9% [76.7–96.1%] to 65.9% [42.5–84.2%]) to a 28.6 (5.8–49.5) percentage-point change in the Xai-Xai district (from 27.6% [16.1–41.0%] to 56.2% [35.5–74.3%]) over the same time period. Even in countries where we estimated an increase in MC coverage for all first and second administrative level units, this heterogeneity persisted. In Uganda, where the estimated national MC coverage increased by 22.9 (15.2–31.3) percentage-points from 2008 to 2017, change in estimated MC prevalence varied from a 3.6 (-4.8, 13.4) percentage-point change in Bukonzo county (from 89.8% [79.8–95.7%] to 93.4% [86.2–97.4%]) to a 40.8 (15.3–61.7) percentage-point change in Masindi county (from 19.6% [8.3–37.0%] to 58.8% [37.8–78.9%]).

### Progress towards MC coverage target

**Fig. 6:**
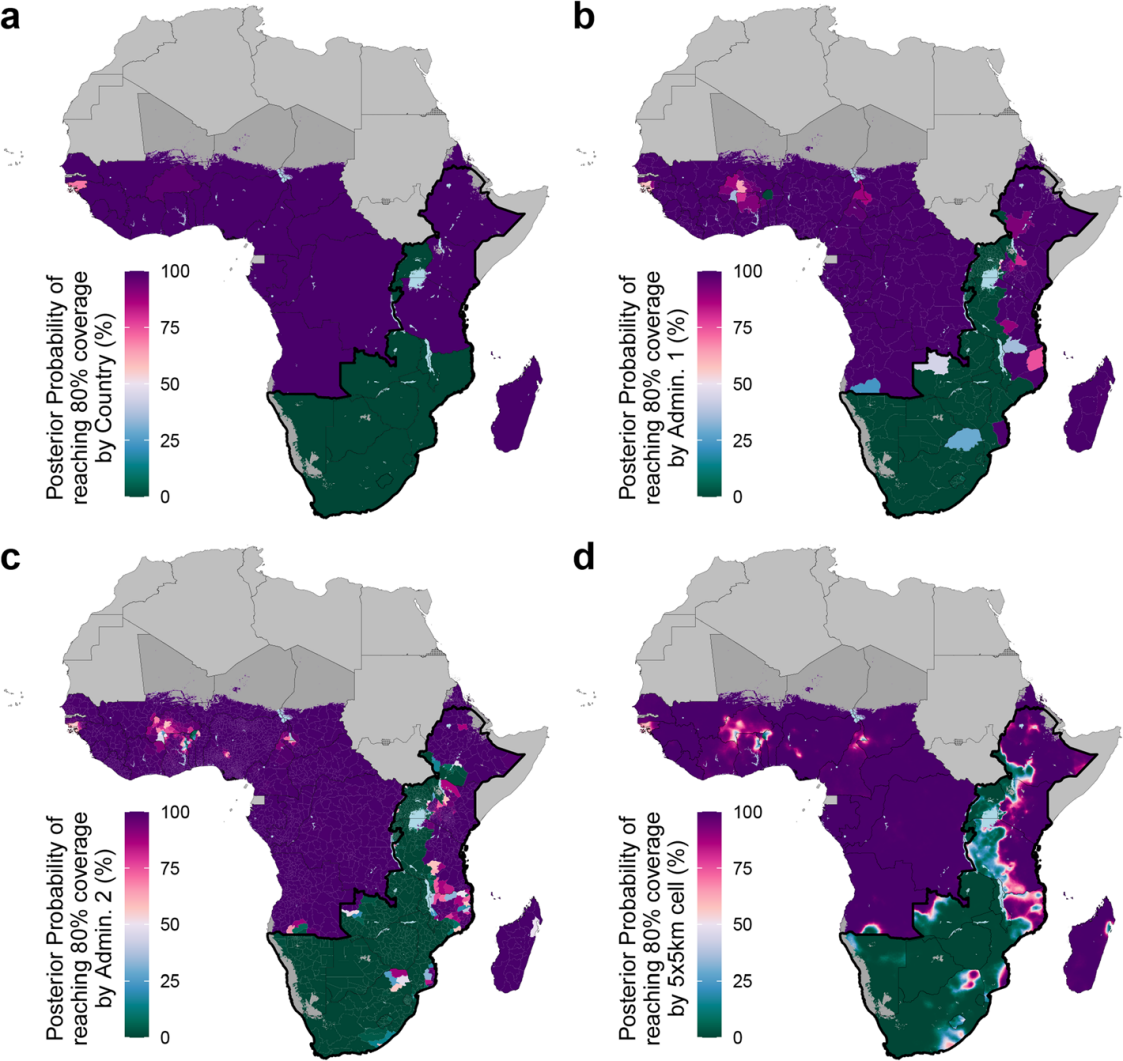
Posterior probability of exceeding 80% male circumcision coverage, adult men ages 15–49, 2017. Posterior probability of exceeding the 80% male circumcision prevalence target among adult men ages 15–49 in 2017 at the country level (**a**), first administrative unit level (**b**), second administrative unit level (**c**), and 5 × 5-km grid-cell level (**d**). Maps reflect administrative boundaries, land cover, lakes, and population. Grid cells with fewer than ten people per 1 × 1-km [39] and classified as “barren or sparsely vegetated” [47] are colored in dark gray. Countries in light gray were not included in the analysis. Outlined by a thick black border are priority countries for VMMC campaigns in southern and eastern Africa, as identified by the WHO and UNAIDS

Despite these increases, we find strong evidence in only three priority countries of having met the goal of 80% MC coverage by 2017 and do not find strong evidence in any country of reaching the 80% coverage target in the collection of administrative units targeted for VMMC. Figure 6 depicts the posterior probability that a given area exceeded the 80% MC coverage target in 2017; high posterior probability indicates substantial evidence that an area met this target, while low posterior probability indicates substantial evidence the area did not meet this target. There was strong evidence that Ethiopia, Kenya, and Tanzania reached the aspirational 80% coverage goal in 2017 (posterior probability > 99.9%) and strong evidence that all other priority countries failed to meet this goal (posterior probability < 0.01%). In some countries, national coverage estimates are less relevant given that VMMC implementation is limited to a regional focus; for example, VMMC implementation in Ethiopia is focused exclusively on the Gambela Region [18]. At subnational levels, the evidence for 80% MC coverage is more mixed, even in countries with strong evidence (posterior probably > 95%) to have reached the national coverage goal. In Ethiopia, 10 (12.7%) of its second administrative level units are unlikely (posterior probability < 5%) to have achieved the coverage target. Conversely, in Mozambique, where there is strong evidence that national MC prevalence did not meet the coverage goal, there is nonetheless strong evidence (posterior probability > 95%) that 33 (25.6%) districts did accomplish this goal in 2017.

## Discussion

This study is a comprehensive and spatially detailed quantification of MC prevalence and the number of circumcised and uncircumcised men living in 38 countries in sub-Saharan Africa from 2000 to 2017. Our estimates highlight meaningful increases in underlying MC coverage in VMMC priority countries but also reveal persistent differences in MC prevalence and the distribution of uncircumcised men across and within those countries. Addressing this geospatial variation is critical for the continued scale-up of VMMC and optimization of MC as a preventative tool to help mitigate the HIV epidemic.

These subnational estimates provide an actionable framework for locally tailored VMMC interventions that acknowledge local epidemiological contexts to reduce HIV transmission. Considering the geographical variation in the HIV epidemic, UNAIDS has repeatedly called for renewed research efforts into the use of spatial analysis in epidemiology and health services research to identify populations in greatest need of prevention services [20, 48]. In accordance with this notion, recent modeling studies have demonstrated that a focused approach to interventions that identify people and locations with low access to HIV prevention strategies like VMMC that are also at high risk of HIV infection is more cost-effective and efficient than a uniform approach in preventing new HIV infections [33–35]. This strategy has already been employed in sub-Saharan Africa: from 2010 to 2014, South Africa allocated provincial resources for VMMC based on population size and epidemiology, which included HIV and MC prevalence [34, 49]. In Tanzania, starting in 2012, geographic information systems were an effective tool for prioritizing VMMC needs in underserved rural communities [50]. This level of geographical precision, however, is not widespread; the WHO and UNAIDS emphasize that most countries lack sufficiently robust data to prioritize provinces or districts according to the potential impact and cost-effectiveness of MC [36]. This analysis provides the spatial precision to fill this gap in data and can be used to identify geographically granular areas where low uptake of MC might be one factor driving local HIV transmission [21]. This, in combination with similarly detailed estimates of HIV prevalence [31], can bolster VMMC programs by highlighting areas where MC coverage is low and HIV burden is high, enabling decision-makers to investigate areas with low coverage and devise locally tailored strategies that continue a trend towards precise and high-impact VMMC interventions [22].

This analysis highlights significant challenges in the scale-up of VMMC and gaps in reaching 80% coverage of MC in priority countries. As opposed to the northern and western regions of sub-Saharan Africa where estimated MC prevalence levels were consistently high, likely due to traditional circumcision practices [51], our estimates highlight spatial and temporal disparities in MC coverage in southern and eastern Africa—especially in high priority countries selected for VMMC scale-up. While all priority countries demonstrated gains in MC coverage since the onset of VMMC national campaigns in 2008, our estimates indicate this improvement has not been uniform between and within countries. Moreover, priority countries Kenya, Tanzania, Mozambique, and Ethiopia all contained areas where estimated MC prevalence decreased from 2008 to 2017, possibly as a result of migration and men aging in and out of the 15–49 age group. The WHO recommends prioritizing VMMC in areas where HIV incidence exceeds the national average, and countries targeting VMMC in groups of men at high risk for HIV or STI infection may explain some local differences in MC coverage [19]. Nonetheless, these disparities are also concurrent with reported implementation delays due to challenges such as financial and human resource limitations, as well as geographical heterogeneity in the desirability and demand for VMMC [16, 37]. Recent studies concluded that 50 to 87% of men in sub-Saharan Africa find VMMC an acceptable form of HIV prevention, highlighting a potential barrier to implementation and uptake as well as a potential source of local differences in MC status [15, 38]. Additional reported barriers include socio-economic factors such as anxiety over the cost of the procedure and a concern for a loss of wages for working-class men, especially in the informal employment sector [52, 53]. The estimated geographical differences may also reflect locations with limited access to VMMC facilities and HIV preventative care [39]. Despite progress, most priority countries were far from achieving 80% coverage in 2017, and none are likely to have achieved this goal in all subnational areas. While the established goal to perform 25 million VMMCs in priority countries by 2020 [20] is laudable, these targets are not sufficient to ensure uniformly high MC coverage at local levels. We estimate that in 2017, there were still 31.6 million uncircumcised men residing in priority countries in sub-Saharan Africa, many of whom were concentrated in a few subnational locations. This analysis complements existing reports of the absolute number of circumcisions performed and provides a framework for tracking progress towards 80% coverage by highlighting areas with high numbers of uncircumcised men and low subnational MC coverage.

While our study focuses on estimates of subnational patterns in MC coverage, VMMC is only one component of a comprehensive HIV prevention package. Additionally, because VMMC provides only partial protection against HIV transmission, care is required to ensure there is no sexual risk compensation among circumcised men—a concern that has been previously raised [54], though other studies have found no evidence of risk compensation [55]. VMMC is projected to avert more than one million new HIV infections by 2030 [2], but VMMC alone is not sufficient to achieve the 2020 global target of fewer than 500,000 new HIV infections annually [20]. In order to be most effective in reducing HIV transmission, VMMC must be combined with other HIV prevention services. VMMC is one of five HIV prevention pillars outlined by UNAIDS, alongside combination prevention for adolescent girls, young women, and their male partners; combination prevention programs for all key populations; comprehensive national condom programs; and rapid introduction of pre-exposure prophylaxis (PrEP) [20]. Countries have already demonstrated the impact of VMMC in this consolidated framework: in Kenya, the dual scale-up of antiretroviral therapy and VMMC was associated with a substantial decline in HIV incidence, and VMMC was suggested to have a direct protective effect [56]. Additionally, continued close integration of VMMC with HIV testing services is crucial given the spatial distribution of the HIV epidemic. Nearly a decade ago, VMMC priority countries were chosen based on low national MC prevalence and high HIV prevalence, and in 2017, a substantial number of areas with low MC coverage still overlapped with areas where HIV prevalence was high [31]. By identifying areas of high HIV prevalence and low MC coverage and continued linkage to HIV testing services, VMMC campaigns could help increase awareness of HIV status among millions of men and boys living in high-prevalence areas and improve access to HIV treatment. Furthermore, additional geospatial mapping of factors that drive the spatial distribution of the HIV epidemic and are components of a comprehensive prevention package such as ART treatment availability, access to PrEP, and condom use could be the next step for reducing HIV burden.

### Limitations

This study is subject to several limitations. First, our analysis is limited by the availability of the underlying data. For our research, we constructed a large database of geo-located MC prevalence data; still, there are gaps in data coverage in both time and space (Supplementary Figs. 1 and 7 in Additional file 1). For example, across VMMC priority countries, the most recent survey used in our analysis ranged from 2013 to 2017. As a result, in priority countries with continued VMMC scale-up but without recent data, we likely underestimate MC prevalence in recent years. Further, over 4 million VMMCs were performed in priority countries in 2018 [18], but currently, there are too few available surveys to reliably extend our analysis to include MC prevalence in 2018. In many VMMC priority countries, however, there are surveys that are either recently completed or currently in the field that will improve our estimates in the future. Second, there are several factors that could impact the quality of the data used in our analysis. Survey data are subject to social desirability bias as well as non-response bias, and the response rate can vary across countries and years. We applied a complete-case analysis that did not adjust for non-response bias; future research could assess the impact of the response rate on estimates of MC prevalence. Our results also rely on self-reported circumcision status that does not distinguish between traditional and medical male circumcision. Past research has found that self-reported MC status may be subject to misreporting errors due to confusion between medical and traditional circumcision practices [57]. Traditional circumcision is often performed in a non-clinical setting, and evidence of its protective effects against HIV transmission is mixed, largely due to discrepancies in defining MC when traditionally performed [27, 29, 30, 58]. Our current analysis provides valuable information on the unmet needs of men who can be reached by VMMC given they have not undergone any form of MC. In the future, distinguishing between non-medical and medical MC would provide a more accurate description of how MC translates to reduced HIV transmission risk. Further, the geographical precision of our data is subject to some error. Most surveys that collect GPS coordinates perform random displacement to safeguard respondent confidentiality; for example, in the Demographic and Health Surveys (DHS), survey clusters are displaced by as much as 2-km for urban clusters, 5-km for rural clusters, and 10-km in 1% of rural clusters [59]. While this can affect a geostatistical model’s predictive power, past research determined that even with GPS displacement, model estimates at a 5 × 5-km resolution are still relatively accurate [60]. Third, while we have attempted to quantify uncertainty in our estimates from various sources, some sources of uncertainty could not be included in the model, such as uncertainty associated with the population estimates. Population estimates from WorldPop are also subject to error, particularly in sparsely populated areas, which may affect the accuracy of our predictions for the number of circumcised and uncircumcised men, especially at fine geographical detail. While WorldPop estimates include censuses data as inputs to their modeling framework [61], depending on timing and data accessibility, estimates may differ from the underlying census measures and may not utilize the most recent census or the most detailed tabulations. Fourth, we use GADM shapefiles to define administrative subdivisions, and differences in administrative divisions between GADM and individual country’s designation of administrative areas may affect the accuracy of results, especially in our estimates of the number of circumcised and uncircumcised men. Fifth, we were unable to include covariates in our model because none of the variables we expect to be most predictive—such as ethnicity, culture, or religion—were available at our spatial or temporal resolution. Future covariates that correlate with ethnicity, culture, or religion would help improve our model predictions in locations and time periods with sparse data coverage. Sixth, our modeling strategy “borrows strength” across space and time to inform estimates in locations and time periods with very small sample sizes and to interpolate in locations and time periods with no directly observed MC prevalence data. While we believe this is generally appropriate, there may be specific locations and times where this methodology breaks down; for example, abrupt differences in MC prevalence in neighboring locations or time periods are unlikely to be reflected in our estimates unless they are directly observed in the underlying MC data and if those data have substantial sample sizes. Finally, our estimates of MC convey only one component of a comprehensive HIV preventative package and only one of many potential drivers of the spatial distribution of the HIV epidemic. Policymakers must take care not to interpret areas of low MC coverage as definite areas of high HIV burden and vice versa; there are many other factors that contribute to the underlying HIV burden at a local scale.

### Future directions

There is ample opportunity to expand this analysis in the future. In addition to including more contemporaneous surveys as they become available, our analysis could also leverage spatially located non-national data sources or data from other research studies. In addition, our current analysis focuses only on one defined age group: males ages 15–49. Past research has shown important variation in the magnitude of impact and cost-effectiveness of VMMC scale-up among different age groups [46] and has indicated that focusing on younger age groups may be the most cost-effective and impactful VMMC intervention [48]. In line with this research, the WHO has called for new coverage targets to be set by age strata as opposed to uniformly set over the entire 15–49 age range [24]. New global targets aligned with the UNAIDS fast track goals aim for 90% MC coverage in men ages 10–29 [19]. While our current analysis focuses on ages 15–49 due to the availability of data and alignment with the original fast track goals, in future analyses, progress towards coverage targets should be monitored by multiple age strata at subnational levels, including younger adolescents. Finally, future analyses that combine estimates of local MC prevalence with information on local HIV incidence could further identify high-risk areas that would benefit from VMMC intervention. At present, the only estimates of HIV burden at the same geographic precision report HIV prevalence, an imperfect measure of the risk of acquiring HIV. In the future, independently constructed estimates of local HIV incidence combined with maps of MC prevalence could provide compelling evidence to identify priority administrative areas for VMMC campaigns.

## Conclusion

In this analysis, we present geographically granular estimates of male circumcision prevalence that can inform efforts to address local HIV prevention needs, especially in high-risk hotspots of HIV transmission in sub-Saharan Africa. While all 14 countries identified as priorities for VMMC by the WHO experienced national increases in MC between 2008 and 2017, half experienced decreases in MC prevalence in some of their subnational areas and none are predicted to meet the 80% MC coverage target across all subnational areas. Our analysis clearly demonstrates that more funding, resources, and accountability are needed in order to reach the proposed coverage goals at all subnational levels by 2020, and our estimates can aid policymakers in resource prioritization decisions. VMMC remains one of the most cost-effective and impactful HIV interventions available, and emphasizing small-scale gaps in coverage and the impact of VMMC services is crucial to maintaining progress towards an end to the HIV/AIDS epidemic.

## Supplementary information

**Additional file 1.**

## Data Availability

Our study follows the Guidelines for Accurate and Transparent Health Estimates Reporting (GATHER). Estimates can be further explored at national, administrative, and local scales through our online visualization tools (https://vizhub.healthdata.org/lbd/circumcision). The source code used to generate estimates, as well as the outputs of the study (including full sets of estimates at the first and second administrative unit levels), are publicly available online via the Global Health Data Exchange (http://ghdx.healthdata.org/record/ihme-data/sub-saharan-africa-male-circumcision-geospatial-estimates-2000-2017). The findings of this study are supported by data available in public online repositories and data publicly available upon request of the data provider; incorporated data sources are shown in Supplementary Table 1. Supplementary Fig. 1 graphs the data input sources by survey, effective sample size, type (point or polygon data), and year for each country modeled. All maps presented in this study are generated by the authors, and no permissions are required to publish them.

## Abbreviations

AIDS: Acquired immunodeficiency syndrome
AIS: AIDS Indicator Survey
CWIQ: Core Welfare Questionnaire Survey
DHS: Demographic and Health Survey
HIV: Human immunodeficiency virus
MC: Male circumcision
MICS: Multiple Indicator Cluster Survey
MSM: Men who have sex with men
PEPFAR: US President’s Emergency Plan for AIDS Relief
PHIA: Population-based HIV Impact Assessment Survey
PrEP: Pre-exposure prophylaxis
UNAIDS: The Joint United Nations Programme on HIV/AIDS
VMMC: Voluntary medical male circumcision
WHO: The World Health Organization

## References

[1] James SL (2018). Global, regional, and national incidence, prevalence, and years lived with disability for 354 diseases and injuries for 195 countries and territories, 1990–2017: a systematic analysis for the Global Burden of Disease Study 2017. Lancet.

[2] World Health Organization. Voluntary medical male circumcision for HIV prevention. 2018. https://www.who.int/hiv/pub/malecircumcision/vmmc-progress-brief-2018/en/. Accessed 29 Apr 2020.

[3] Gray RH (2007). Male circumcision for HIV prevention in men in Rakai, Uganda: a randomised trial. Lancet.

[4] Bailey RC (2007). Male circumcision for HIV prevention in young men in Kisumu, Kenya: a randomised controlled trial. Lancet.

[5] Auvert B (2005). Randomized, controlled intervention trial of male circumcision for reduction of HIV infection risk: the ANRS 1265 Trial. PLoS Med.

[6] Gray R (2012). The effectiveness of male circumcision for HIV prevention and effects on risk behaviors in a posttrial follow-up study. AIDS Lond Engl.

[7] Auvert B (2013). Association of the ANRS-12126 male circumcision project with HIV levels among men in a south African township: evaluation of effectiveness using cross-sectional surveys. PLoS Med.

[8] Wamai RG, Morris BJ, Bailey RC, Klausner JD, Boedicker MN (2015). Male circumcision for protection against HIV infection in sub-Saharan Africa: the evidence in favour justifies the implementation now in progress. Glob Public Health.

[9] Gray R, Male H (2019). Circumcision for HIV and STI prevention: a reflection. Clin Chem.

[10] Wamai RG (2011). Male circumcision for HIV prevention: current evidence and implementation in sub-Saharan Africa. J Int AIDS Soc.

[11] Wamai RG (2012). Criticisms of African trials fail to withstand scrutiny: male circumcision does prevent HIV infection. J Law Med.

[12] Lei JH (2015). Circumcision status and risk of HIV acquisition during heterosexual intercourse for both males and females: a meta-analysis. PLoS One.

[13] Davis SM, et al. Progress in voluntary medical male circumcision for HIV prevention supported by the US President’s Emergency Plan for AIDS Relief through 2017: longitudinal and recent cross-sectional programme data. BMJ Open. 2018;8(8):e021835. 10.1136/bmjopen-2018-021835 PMC6120649 30173159

[14] Reed JB (2012). Voluntary medical male circumcision: an HIV prevention priority for PEPFAR. J Acquir Immune Defic Syndr.

[15] Grund JM (2017). Association between male circumcision and women’s biomedical health outcomes: a systematic review. Lancet Glob Health.

[16] Pintye J, Baeten JM (2019). Benefits of male circumcision for MSM: evidence for action. Lancet Glob Health.

[17] UNAIDS. Joint strategic action framework to accelerate the scale-up of voluntary medical male circumcision for HIV prevention in eastern and southern Africa, 2012–2016. 2011. https://www.who.int/hiv/pub/strategic_action2012_2016/en/. Accessed 5 June 2019.

[18] World Health Organization. Remarkable progress in the scale up of voluntary medical male circumcision as an HIV prevention intervention in 15 ESA countries. 2019. https://www.who.int/publications-detail/voluntary-medical-male-circumcision-progress-brief-2019. Accessed 13 Mar 2020.

[19] World Health Organization. A framework for voluntary medical male circumcision: effective HIV prevention and a gateway to improved adolescent boys’ and men’s health in eastern and southern Africa by 2021. https://apps.who.int/iris/bitstream/handle/10665/246234/WHO-HIV-2016.17-eng.pdf?sequence=1. Accessed 29 Apr 2020.

[20] UNAIDS. Global AIDS update 2018: miles to go. 2018. https://www.unaids.org/sites/default/files/media_asset/miles-to-go_en.pdf. Accessed 4 June 2019.

[21] Cuadros DF, Branscum AJ, Miller FD, Awad SF, Abu-Raddad LJ (2015). Are geographical ‘cold spots’ of male circumcision driving differential HIV dynamics in Tanzania?. Front Public Health.

[22] Akullian A, Onyango M, Klein D, Odhiambo J, Bershteyn A. Geographic coverage of male circumcision in western Kenya. Medicine (Baltimore). 2017;96. 10.1097/MD.0000000000005885 PMC5266192 28079830

[23] Moses S (1990). Geographical patterns of male circumcision practices in Africa: association with HIV seroprevalence. Int J Epidemiol.

[24] Weiss HA, Quigley MA, Hayes RJ (2000). Male circumcision and risk of HIV infection in sub-Saharan Africa: a systematic review and meta-analysis. AIDS Lond. Engl..

[25] Morris BJ (2016). Estimation of country-specific and global prevalence of male circumcision. Popul Health Metrics.

[26] Drain PK, Halperin DT, Hughes JP, Klausner JD, Bailey RC (2006). Male circumcision, religion, and infectious diseases: an ecologic analysis of 118 developing countries. BMC Infect Dis.

[27] Wilcken A, Keil T, Dick B (2010). Traditional male circumcision in eastern and southern Africa: a systematic review of prevalence and complications. Bull World Health Organ.

[28] World Health Organization. Traditional male circumcision among young people. 2009. https://www.who.int/hiv/pub/malecircumcision/traditional_mc/en/. Accessed 13 Mar 2020.

[29] Shaffer DN (2007). The protective effect of circumcision on HIV incidence in rural low-risk men circumcised predominantly by traditional circumcisers in Kenya: two-year follow-up of the Kericho HIV Cohort Study. J Acquir Immune Defic Syndr.

[30] Bailey RC, Egesah O, Rosenberg S (2008). Male circumcision for HIV prevention: a prospective study of complications in clinical and traditional settings in Bungoma, Kenya. Bull World Health Organ.

[31] Dwyer-Lindgren L (2019). Mapping HIV prevalence in sub-Saharan Africa between 2000 and 2017. Nature.

[32] Anderson S-J (2014). Maximising the effect of combination HIV prevention through prioritisation of the people and places in greatest need: a modelling study. Lancet.

[33] Kripke K (2016). Voluntary medical male circumcision for HIV prevention in Malawi: modeling the impact and cost of focusing the program by client age and geography. PLoS One.

[34] Kripke K (2016). Cost and impact of voluntary medical male circumcision in South Africa: focusing the program on specific age groups and provinces. PLoS One.

[35] Kripke K (2016). The economic and epidemiological impact of focusing voluntary medical male circumcision for HIV prevention on specific age groups and regions in Tanzania. PLoS One.

[36] World Health Organization. Male circumcision for HIV prevention: models to inform fast tracking voluntary medical male circumcision in HIV combination prevention. 2016. https://www.who.int/hiv/pub/malecircumcision/fast-tracking-male-circumcision/en/. Accessed 4 June 2019.

[37] Ledikwe JH (2014). Scaling-up voluntary medical male circumcision – what have we learned?. HIVAIDS Auckl NZ.

[38] Global Health Data Exchange. *GHDx* http://ghdx.healthdata.org. Accessed 29 Apr 2020.

[39] WorldPop Population Dataset. https://www.worldpop.org/project/categories?id=3 (Accessed 7 July 2017).

[40] Osgood-Zimmerman A (2018). Mapping child growth failure in Africa between 2000 and 2015. Nature.

[41] Lim SS, Stein DB, Charrow A, Murray CJ (2008). Tracking progress towards universal childhood immunisation and the impact of global initiatives: a systematic analysis of three-dose diphtheria, tetanus, and pertussis immunisation coverage. Lancet.

[42] Graetz N (2018). Mapping local variation in educational attainment across Africa. Nature.

[43] Reiner RC (2018). Variation in childhood diarrheal morbidity and mortality in Africa, 2000–2015. N Engl J Med.

[44] GADM database of global administrative areas. https://gadm.org/ (Accessed 6 May 2018).

[45] Rue H, Martino S, Chopin N (2009). Approximate Bayesian inference for latent Gaussian models by using integrated nested Laplace approximations. J R Stat Soc Ser B Stat Methodol.

[46] The R-INLA project. *R-INLA* http://www.r-inla.org/. Accessed 25 July 2019.

[47] Land Processes Distributed Active Archive Center. Combined MODIS 5.1 dataset. MCD12Q1 | LP DAAC :: NASA Land Data Products and Services. https://lpdaac.usgs.gov/dataset_discovery/modis/modis_products_table/mcd12q1/. Accessed 1 June 2017.

[48] UNAIDS. On the Fast-Track to end AIDS by 2030: Focus on location and population. 2015. https://www.unaids.org/en/resources/documents/2015/FocusLocationPopulation. Accessed 13 Mar 2020.

[49] McGillen JB, Anderson S-J, Dybul MR, Hallett TB (2016). Optimum resource allocation to reduce HIV incidence across sub-Saharan Africa: a mathematical modelling study. Lancet HIV.

[50] Mahler H (2015). Covering the last kilometer: using GIS to scale-up voluntary medical male circumcision services in Iringa and Njombe Regions, Tanzania. Glob Health Sci Pract.

[51] Lawal TA, Olapade-Olaopa EO (2017). Circumcision and its effects in Africa. Transl Androl Urol.

[52] Herman-Roloff A, Otieno N, Agot K, Ndinya-Achola J, Bailey RC (2011). Acceptability of medical male circumcision among uncircumcised men in Kenya one year after the launch of the national male circumcision program. PLoS One.

[53] Westercamp N, Bailey RC (2007). Acceptability of male circumcision for prevention of HIV/AIDS in sub-Saharan Africa: a review. AIDS Behav.

[54] Kibira SPS, Sandøy IF, Daniel M, Atuyambe LM, Makumbi FE (2016). A comparison of sexual risk behaviours and HIV seroprevalence among circumcised and uncircumcised men before and after implementation of the safe male circumcision programme in Uganda. BMC Public Health.

[55] Shi C-F, Li M, Dushoff J. Evidence that promotion of male circumcision did not lead to sexual risk compensation in prioritized sub-Saharan countries. PLoS One. 2017;12. 10.1371/journal.pone.0175928 PMC5404849 28441458

[56] Borgdorff MW (2018). HIV incidence in western Kenya during scale-up of antiretroviral therapy and voluntary medical male circumcision: a population-based cohort analysis. Lancet HIV.

[57] Thomas AG (2011). Voluntary medical male circumcision: a cross-sectional study comparing circumcision self-report and physical examination findings in Lesotho. PLoS One.

[58] Maffioli EM. Is traditional male circumcision effective as an HIV prevention strategy? Evidence from Lesotho. PLoS ONE. 2017;12(5). 10.1371/journal.pone.0177076 PMC5428932 28498835

[59] Burgert CR, Colston J, Roy T, Zachary B. Geographic displacement procedure and georeferenced data release policy for the Demographic and Health Surveys. 2013. http://www.dhsprogram.com/publications/publication-SAR7-Spatial-Analysis-Reports.cfm. Accessed 29 Apr 2020.

[60] Gething P, Tatem A, Bird T, Burgert-Brucker CR (2015). Creating spatial interpolation surfaces with DHS data.

[61] Stevens FR, Gaughan AE, Linard C, Tatem AJ (2015). Disaggregating census data for population mapping using random forests with remotely-sensed and ancillary data. PLoS One.

