# Supplementary material for: Mapping male circumcision for HIV prevention efforts in sub-Saharan Africa
Source: BMC Med. 2020 Jul 7;18:189. doi: 10.1186/s12916-020-01635-5 (PMC7339571; doi:10.1186/s12916-020-01635-5)
Supplement: Supplementary file 1 — Additional file 1. [file 12916_2020_1635_MOESM1_ESM.docx]

Additional file 1: Mapping male circumcision for HIV prevention efforts in sub-Saharan Africa

Table of Contents

[1 Compliance with the Guidelines for Accurate and Transparent Health Estimates Reporting (GATHER) 2](#_Toc35604327)

[2 Data 4](#_Toc35604328)

[2.1 Prevalence surveys 4](#_Toc35604329)

[2.2 Age cross-walk 4](#_Toc35604330)

[2.3 Polygon resampling 5](#_Toc35604331)

[2.4 Administrative boundaries 6](#_Toc35604332)

[2.5 Gridded populations 6](#_Toc35604333)

[3 Statistical model 6](#_Toc35604334)

[3.1 Geostatistical model 6](#_Toc35604335)

[3.2 Model validation 10](#_Toc35604336)

[3.3 Post estimation 11](#_Toc35604337)

[References 13](#_Toc35604338)

[Supplementary Figures 14](#_Toc35604339)

[Supplementary Figure 1: Male circumcision data availability. 14](#_Toc35604340)

[Supplementary Figure 2: Analytic process overview. 15](#_Toc35604341)

[Supplementary Figure 3: Modelling regions. 16](#_Toc35604342)

[Supplementary Figure 4: Estimated male circumcision prevalence, ages 15–49, 2000. 17](#_Toc35604343)

[Supplementary Figure 5: Estimated male circumcision prevalence, ages 15–49, 2005. 18](#_Toc35604344)

[Supplementary Figure 6: Estimated male circumcision prevalence, ages 15–49, 2010. 19](#_Toc35604345)

[Supplementary Figure 7: National data and estimates. 20](#_Toc35604346)

[Supplementary Figure 8: Space mesh for geostatistical models. 21](#_Toc35604347)

[Supplementary Tables 22](#_Toc35604348)

[Supplementary Table 1: Male circumcision survey data. 22](#_Toc35604349)

[Supplementary Table 2: Surveys excluded from this analysis. 43](#_Toc35604350)

[Supplementary Table 3: Data requiring age cross-walk. 44](#_Toc35604351)

[Supplementary Table 4: Number and designated name for first and second administrative level units 45](#_Toc35604352)

[Supplementary Table 5: Validation metrics. 47](#_Toc35604353)

[Supplementary Table 6: Prior sensitivity analysis results. 48](#_Toc35604354)

[Supplementary Table 7: Fitted model parameters. 49](#_Toc35604355)

# 1 Compliance with the Guidelines for Accurate and Transparent Health Estimates Reporting (GATHER)

| Item # | Checklist item | Description of Compliance |
| --- | --- | --- |
| Objectives and funding | | |
| 1 | Define the indicator(s), populations (including age, sex, and geographical entities), and time period(s) for which estimates were made. | Manuscript: Background, Methods (Data collection); Appendix: section 2.1 Prevalence surveys |
| 2 | List the funding sources for the work. | Manuscript: Declarations (Funding) |
| Data Inputs | | |
| *For all data inputs from multiple sources that are synthesised as part of the study:* | | |
| 3 | Describe how the data were identified and how the data were accessed. | Manuscript: Methods (Data collection); Appendix: section 2.1 Prevalence surveys |
| 4 | Specify the inclusion and exclusion criteria. Identify all ad-hoc exclusions. | Manuscript: Methods (Data collection); Appendix: section 2.1 Prevalence surveys; Supplementary Table 2 |
| 5 | Provide information on all included data sources and their main characteristics. For each data source used, report reference information or contact name/institution, population represented, data collection method, year(s) of data collection, sex and age range, diagnostic criteria or measurement method, and sample size, as relevant. | Supplementary Tables 1 and 2 |
| 6 | Identify and describe any categories of input data that have potentially important biases (e.g., based on characteristics listed in item 5). | Manuscript: Discussion (Limitations) |
| *For data inputs that contribute to the analysis but were not synthesised as part of the study:* | | |
| 7 | Describe and give sources for any other data inputs. | Appendix: section 2.4 Administrative borders and section 2.5 Gridded populations |
| *For all data inputs:* | | |
| 8 | Provide all data inputs in a file format from which data can be efficiently extracted (e.g., a spreadsheet rather than a PDF), including all relevant meta-data listed in item 5. For any data inputs that cannot be shared because of ethical or legal reasons, such as third-party ownership, provide a contact name or the name of the institution that retains the right to the data. | Available through GHDx link (upon publication) |
| Data analysis | | |
| 9 | Provide a conceptual overview of the data analysis method. A diagram may be helpful. | Manuscript: Methods (Data analysis); Supplementary Figure 2 |
| 10 | Provide a detailed description of all steps of the analysis, including mathematical formulae. This description should cover, as relevant, data cleaning, data pre-processing, data adjustments and weighting of data sources, and mathematical or statistical model(s). | Manuscript: Methods; Appendix: section 2 Data and section 3 Statistical model |
| 11 | Describe how candidate models were evaluated and how the final model(s) were selected. | Appendix: section 3.2 Model validation |
| 12 | Provide the results of an evaluation of model performance, if done, as well as the results of any relevant sensitivity analysis. | Appendix: section 3.2 Model validation |
| 13 | Describe methods for calculating uncertainty of the estimates. State which sources of uncertainty were, and were not, accounted for in the uncertainty analysis. | Manuscript: Methods; Appendix: section 3.2 Model validation and section 3.3 Post estimation |
| 14 | State how analytic or statistical source code used to generate estimates can be accessed. | Available through GHDx link (upon publication) |
| Results and Discussion | | |
| 15 | Provide published estimates in a file format from which data can be efficiently extracted. | Available through GHDx link (upon publication) |
| 16 | Report a quantitative measure of the uncertainty of the estimates (e.g., uncertainty intervals). | Manuscript: Results and Figure 2 |
| 17 | Interpret results in light of existing evidence. If updating a previous set of estimates, describe the reasons for changes in estimates. | Manuscript: Discussion |
| 18 | Discuss limitations of the estimates. Include a discussion of any modelling assumptions or data limitations that affect interpretation of the estimates. | Manuscript: Discussion |

# 2 Data

## 2.1 Prevalence surveys

#### 2.1.1 Data identification strategy

We identified male circumcision (MC) prevalence data through a review of major survey series in sub-Saharan Africa, including: Demographic and Health Surveys (DHS); AIDS Indicator Surveys (AIS); Multiple Indicator Cluster Surveys (MICS); Core Welfare Indicators Questionnaire Surveys (CWIQ); Population-based HIV Impact Assessment Surveys (PHIA); Reproductive Health Surveys (RHS); Living Standards Measurement Surveys (LSMS); and other, country-specific surveys (CS). We supplemented this initial list of surveys with country-specific surveys identified in the Global Health Data Exchange^1^ and with a cross-check of all surveys extracted for our previous work on HIV prevalence^2^ (Supplementary Table 1). To be included in the analysis, we required each survey to ask male respondents if they were circumcised, sample from the adult male population ages 15–49, and contain geographical information at a more granular resolution than the country level. In the case that a survey did not contain any microdata, we included reports that provided estimates for the 15–49 age group and included sample size or uncertainty intervals.

#### 2.1.2 Data processing for microdata

To prepare survey microdata for analysis, we first subset the data to males age 15 to 49. We then dropped rows missing a response for whether or not they were circumcised or missing a survey weight (2.8% of all observations), and rows missing geographical information or with geographical coordinates located more than 10 km outside of the country border (1.2% of all observations); coordinates within 10 km of the country border were snapped to be approximately 1 km inside the nearest border of the specified country.

We then aggregated the individual-level microdata to calculate MC prevalence at the finest possible spatial resolution available, ideally a latitude and longitude pair representing the location of the survey cluster (point-level data). Where point-level referencing was not available, we geo-located survey microdata to the smallest geographical area (polygon) possible. Individual-level sample weights were used when calculating prevalence, and the effective sample size for each prevalence estimate was estimated *via* the Kish approximation,^3^ which accounts for differences in the underlying selection probability within a sample. The interview date for each specific location was calculated as the median of the individual-level interview dates.

#### 2.1.3 Data processing for reports

In situations where individual-level microdata were not available, we utilized summary reports. We used the median months of the reported data collection periods (ranging from 2 to 13 months in length) as the interview dates to align with the extracted microdata. We adjusted reported sample sizes by multiplying by the median design effect (ratio of effective sample size to observed sample size) calculated in the microdata as described above. For prevalence estimates provided for an age range other than 15–49 years, we cross-walked the prevalence to estimate prevalence for the 15–49 age range (see section 2.2 for details).

## 2.2 Age cross-walk

In some instances, MC prevalence estimates extracted from survey reports referred to age ranges other than the standard 15–49 age range, which we denote below as $A_{1}-A_{2}$ where $A_{1}\neq15$ and/or $A_{2}\neq49$. In these instances, we used linear regression to transform the estimates of MC prevalence from the reported age range $A_{1}-A_{2}$ to MC prevalence for the standard 15–49 age range. The methods for the age-cross-walk are consistent with those previously used in the geospatial modelling of HIV.^2^

To do this, for each $A_{1}-A_{2}$ we first identified surveys with microdata available that spanned both the $A_{1}-A_{2}$ and 15–49 age ranges, i.e., ${\mathrm{minimum}(A}_{1},15)$-${\mathrm{maximum}(A}_{2},49)$, and produced MC prevalence estimates for both age ranges by first administrative level units. Then, for each non-standard age range, we used these data to fit the following linear regression model:

$$\mathrm{logit}\left( p_{15-49} \right)= \beta_{0}+ \beta_{1}\cdot logit\left( p_{A_{1}-A_{2}} \right)+ \epsilon$$

where:

- $p_{15-49}$ is the observed MC prevalence among individuals age 15–49 years;
- $p_{A_{1}-A_{2}}$ is the observed MC prevalence among individuals age $A_{1}-A_{2}$ years;
- $\beta_{0}$ is an intercept;
- $\beta_{1}$ is the slope on $\mathrm{logit}\left( p_{A_{1}-A_{2}} \right)$;
- $\epsilon$is a normally distributed error term with mean 0.

In this model, a logit transform was applied to prevalence to ensure the predicted prevalence was restricted to between 0 and 1. For values that were equal to 0 and 1, a transformation was employed to preserve the shape of the logistic curve. For these values, an 𝜀 was selected to be half of the smallest non-zero value, and all instances of 0 were replaced with 𝜀 and instances of 1 replaced with one minus epsilon (1− 𝜀). Linear regression was chosen over more complex algorithms due to the high proportion of variance explained with this relatively simple method (R^2^ ranged from 0.92 to 0.99).

Uncertainty in prevalence for ages 15–49 as a result of this cross-walk was estimated by simulating 1,000 draws of the slope, intercept, and error term from the fitted linear model, and using these to construct 1,000 draws of MC prevalence among ages 15–49.^4^ Then, for each draw of MC prevalence, we simulated a random draw from a binomial distribution with *p* equal to prevalence from this draw and *N* equal to the reported sample size and then divided this simulated count by the reported sample size to again calculate MC prevalence. These draws of MC prevalence reflected both the sampling uncertainty as well as the additional uncertainty introduced by cross-walking from non-standard age ranges to 15–49. Finally, a new effective sample size was calculated by finding the variance of the prevalence draws and employing the relationship between variance and sample size of a binomial distribution. This new sample size reflected our confidence in the estimate of MC prevalence for ages 15–49 and is a function of the uncertainty in our linear model, the original sample size, and level of MC prevalence. Surveys where this age cross-walk was required are listed in Supplementary Table 3.

## 2.3 Polygon resampling

Wherever possible, we matched MC prevalence data to a specific latitude and longitude. In instances where this was not possible, we matched data to the smallest areal unit (termed a polygon). In most instances, these polygons represented administrative units. The statistical model we employed requires point-referenced data, so data matched to polygons were resampled to generate pseudo-point data based on the underlying population distribution within the polygon. The technical descriptions of methods for the resampling are consistent with those previously used in the geospatial modelling of HIV.^2^

In this framework, we randomly sampled 10,000 locations among grid cells for each polygon-level observation with probability proportional to the grid cell population. We specified grid cells as contained within a polygon if their centroid fell within the geographical boundary. We then performed k-means clustering (with k set to 1 per 40 grid cells) on the sampled points to generate a reduced set of locations based on the k-means cluster centroids to be used in modelling. We assigned weights to each pseudo-point proportional to the number of sampled points contained in each of the k-means clusters, which translated to the number of sampled points divided by 10,000. We assigned each pseudo-point generated by this process the MC prevalence observed for the polygon as a whole, and a sample size equal to the sample size for the polygon as a whole multiplied by the weight derived for each point.

## 2.4 Administrative boundaries

For this analysis we used the Database of Global Administrative Areas (GADM) shapefiles^5^ to define boundaries for countries and their first and second administrative level units. Minor adjustments to these shapefiles were made where names were missing in the original shapefiles or where more accurate boundary information was identified in other sources.

## 2.5 Gridded populations

The gridded population data used for this analysis were obtained from WorldPop.^6^ Because WorldPop provides data at a 1 × 1-km spatial resolution at five-year intervals for men ages 15–49, we first aggregated the raster by taking the sum of the finer raster to produce the total population at a 5 × 5-km resolution. To resolve the difference in temporal resolution, we filled the intervening year with an exponential growth rate model to produce a raster of annual population counts.

# 3 Statistical model

## 3.1 Geostatistical model

The technical descriptions of methods for the underlying geostatistical model are consistent with those previously used in the geospatial modelling of HIV.^2^

#### 3.1.1 Model description

We modelled the number of circumcised men ($Y_{i,t}$) among a sample ($N_{i,t}$) in location *i* and year *t* as a binomial variable. This model specified logit-transformed circumcision prevalence ($p_{i,t}$) as a linear combination of a regional intercept ($\beta_{0}$), regional fixed effect on year ($\beta_{1}t$), country random effect ($\gamma_{c\left[ i \right]}$), spatially- and temporally-correlated random effect ($Z_{i,t}$), and an uncorrelated error term or nugget effect ($\epsilon_{i,t}$). The equations for the spatially and temporally explicit generalized linear mixed effects model are shown below:

$$Y_{i,t}\sim Binomial\left( p_{i,t}, N_{i,t} \right)$$

$$\mathrm{logit}\left( p_{i,t} \right)= \beta_{0}+{\beta_{1}t+ \gamma_{c\left[ i \right]}+ Z}_{i,t}+ \epsilon_{i,t}$$

$$\gamma_{c\left[ i \right]}\sim Normal\left( 0, \sigma_{country}^{2} \right)$$

$$Z_{i,t}\sim GP(0, \Sigma_{space} \bigotimes\Sigma_{time})$$

$$\epsilon_{i,t}\sim Normal\left( 0, \sigma_{nugget}^{2} \right)$$

where:

- $N_{i,t}$ and $Y_{i,t}$ are the number of men sampled and the number of men who were circumcised among those sampled, respectively, in location *i* and year *t*;
- $p_{i,t}$ is the underlying male circumcision prevalence in location *i* and year *t*;
- $\beta_{0}$ is an intercept;
- $\beta_{1}$ is a fixed-effect on year *t* (coded with *t* = 1 for year 2000, *t* = 2 for year 2001, etc.);
- $\gamma_{c\left[ i \right]}$ is a country-level random effect for country *c* containing location *i*;
- $Z_{i,t}$ is a spatially and temporally correlated random effect for location ­­*i* and year *t*;
- $\epsilon_{i,t}$ is an independent and identically distributed random effect for location ­­*i* and year *t*.

Descriptively, the intercept captures the regional mean level of MC prevalence in 1999 and the fixed-effect on year captures the general regional trend in MC over time. The country random effect captures additional variation between countries while the spatially and temporally correlated random effect captures additional variation by location (within and between countries) and time that varies smoothly in space and time. Finally, the uncorrelated random effect (or nugget effect) captures any additional, non-structured variation by location and time. We did not include covariates for this analysis because covariates that we expected to be predictive, such as ethnicity, culture, or religion, were not available at the same spatial and temporal resolution as our input data.

We modelled the spatially and temporally correlated random effect ($Z_{i,t}$) as a Gaussian process with mean 0 and a covariance matrix given by the Kronecker product of a spatial Matérn covariance function ($\Sigma_{space}$) and a temporal first-order autoregressive (AR1) covariance function ($\Sigma_{time}$). The Matérn covariance function is given by:

$$\Sigma_{space}=\sigma^{2}\frac{2^{1-\nu}}{\Gamma\left( \nu\right)}\times\left( \kappa\boldsymbol{D} \right)^{\nu}\times K_{\nu}\left( \kappa\boldsymbol{D} \right)$$

In this analysis $\nu$ (the smoothness parameter) was fixed at 1. For $Z_{i,t}$ a penalized complexity (PC) prior was used for the Matérn covariance function and specified *via* two hyper-parameters: the spatial range, $\rho_{s}$ (where $\rho_{s}=\sqrt{8\nu}/\kappa$ and is equal to the distance, measured in relation to the true radius of the earth [6371 km], at which correlation is approximately 0.1; the subscript *s* for space is used as to not confuse with the temporal correlation parameter), and marginal standard deviation, σ. PC priors shrink towards a more simplistic base model – in this case, one where the marginal variance is 0 and the spatial range is infinite – and are specified *via* setting the tail probabilities on each hyper-parameter.^7,8^ We followed the guidance provided by Fugulstad *et al.*, who recommend selecting priors that satisfy $P\left( s > s_{0} \right)=0.05$ and $P\left( \rho_{s}< \rho_{s_{0}} \right)=0.05$, where $s_{0}$ is between 2.5 to 40 times the expected true marginal standard deviation and $\rho_{s_{0}}$ is between $1/{10}$ to $1/{2.5}$ of the expected true range.^8^ Specifically, we set:

$$s_{0}=10; P\left( s > s_{0} \right)=0.05$$

$$\rho_{s_{0}}=0.01; P\left( \rho_{s}< \rho_{s_{0}} \right)=0.05$$

In addition, for $Z_{i,t}$, the AR1 covariance function is associated with a temporal correlation parameter $\rho$ (not be confused with $\rho_{s}$). We used the following hyper-prior in this case, which corresponds to a prior mean of 0.76 with a 95% range of -0.17 to 0.97 for $\rho$:

$$log((1+\rho)/(1-\rho))\sim Normal(2, {1.2}^{2})$$

PC priors were also used for the standard deviation of $\gamma_{c\left[ i \right]}$ and $\epsilon_{i,t}$ and were set to:

$$\sigma_{{country}_{0}}=10; P\left( \sigma_{country}> \sigma_{{country}_{0}} \right)=0.05$$

$$\sigma_{{nugget}_{0}}=3; P\left( \sigma_{nugget}> \sigma_{{nugget}_{0}} \right)=0.05$$

Finally, the priors for the intercept and the fixed effect on time were set to:

$$\beta_{0}\sim\mathrm{Normal}\left( 0, 3^{2} \right)$$

$$\beta_{1}\sim\mathrm{Normal}\left( 0, 3^{2} \right)$$

#### 3.1.2 Prior sensitivity analysis

We undertook a sensitivity analyses to assess the impact of the hyper-priors for all random effects on the model predictions. We considered five alternate models with different hyper-prior specifications, varying how general or informative each model hyper-prior was to assess its impact on our predictions.

- Model 1 (Less informative PC priors):

In this model, we retained the PC priors for $\rho_{s}$ and σ, but made these hyper-priors less informative by specifying a larger value of the standard deviation and smaller value of the spatial range when setting the tail probabilities:

$$s_{0}=20; P\left( s > s_{0} \right)=0.05$$

$$\rho_{s_{0}}=0.005; P\left( \rho_{s}< \rho_{s_{0}} \right)=0.05$$

We similarly updated the PC priors for the standard deviation of $\gamma_{c\left[ i \right]}$ and $\epsilon_{i,t}$:

$$\sigma_{{country}_{0}}=20; P\left( \sigma_{country}> \sigma_{{country}_{0}} \right)=0.05$$

$$\sigma_{{nugget}_{0}}=6; P\left( \sigma_{nugget}> \sigma_{{nugget}_{0}} \right)=0.05$$

The hyper-prior for the temporal correlation, $\rho$, was the same as our selected model.

- Model 2 (Less informative PC priors with informative $\rho$):

This model has the same less-informative PC priors as Model 1 on the spatial parameters and other random effects, but the hyper-prior for the temporal correlation parameter $\rho$ was adjusted to be more heavily weighted toward a high temporal correlation:

$$log((1+\rho)/(1-\rho))\sim Normal(4, {1.2}^{2})$$

This hyper-prior corresponds to a prior mean of 0.96 with a 95% range of 0.68 to 1 for $\rho$.

- Model 3 (INLA default):

This model used the default hyper-priors in INLA implemented in the function inla.spde2.matern()^9^. These hyper-priors are defined in terms of $\tau$ (where $=\sqrt{1/(4\pi\kappa^{2}\sigma^{2})}$ ) and $\kappa$, and were set as:

$$\theta_{1}=log \left( \tau\right)\sim\mathrm{Normal}\left( \mu_{\theta_{1}}, \sigma_{\theta_{1}}^{2} \right)$$

$$\theta_{2}=log \left( \kappa\right)\sim\mathrm{Normal}\left( \mu_{\theta_{2}}, \sigma_{\theta_{2}}^{2} \right)$$

with $\mu_{\theta_{1}}$, $\sigma_{\theta_{1}}$, $\mu_{\theta_{2}}$, and $\sigma_{\theta_{2}}$automatically determined by INLA.

INLA default hyper-priors were also used for the precision parameter of the remaining random effects:

$$1/{\sigma_{country}^{2}}\sim\mathrm{gamma}\left( \mathrm{rate}=1, \mathrm{shape}=0.00005 \right)$$

$$1/{\sigma_{nugget}^{2}}\sim\mathrm{gamma}\left( \mathrm{rate}=1, \mathrm{shape}=0.00005 \right)$$

The hyper-prior for the temporal correlation parameter, $\rho$, was the same as our selected model.

- Model 4 (INLA default with informative $\rho$):

This model had the same hyper-priors for $\tau$, $\kappa$, $\sigma_{country}^{2}$, and $\sigma_{nugget}^{2}$ as Model 3, but the more informative hyper-prior for the temporal correlation parameter, $\rho$, described for Model 2.

- Model 5 (default PC prior with informative $\rho$):

This model used the same PC priors as our selected model but used the more informative hyper-prior for the temporal correlation parameter, $\rho$, described for Model 2.

These six models (five alternative models and our base model) were fit for MC prevalence to enable comparison between model predictions. The predictions from each of the five alternative models were highly correlated at the first and second administrative levels, with the pairwise correlation above 0.999 (Supplementary Table 6). Additionally, in all cases there was 100% overlap in the uncertainty intervals between the selected model and any alternate model. The mean absolute difference between predictions was also generally low. These comparisons suggest that the predictions are relatively robust to different hyper-prior specifications.

#### 3.1.3 Model fitting and prediction

We fit this model in R-INLA^10^ using the stochastic partial differential equations (SPDE)^9^ approach to approximate the continuous spatial-temporal Gaussian random field ($Z_{i,t}$). Using a simplified polygon boundary, we created a finite elements mesh for the SPDE approximation to the Gaussian process regression (Supplementary Figure 7). We used a spatial mesh that was constructed on the S^2^ domain which allowed distance to be calculated along a sphere instead of using Euclidean distance between latitude and longitude coordinates. We set the minimum triangle edge length to 20 kilometers, the maximum triangle length to 500 kilometers, with the mesh extending 500 kilometers past the region’s boundary. Estimated model parameters are listed in Supplementary Table 7.

Due to computational constraints and to allow for regional differences in the strength of spatial and temporal auto-correlation in MC prevalence, separate models were fit for four regions (Supplementary Figure 3). Specifically, we used the regional classifications for sub-Saharan Africa from the Global Burden of Disease (GBD) study^11^ which group countries by location and epidemiological profile. For each regional sub-model, data from the modelled region as well as data within a one-degree buffer of the region’s boundary were included (in order to minimize regional edge effects). We removed countries from our analysis if we did not find survey data during the years of our analysis, and this resulted in several modifications to the GBD classification: we removed Equatorial Guinea from the Central sub-Saharan Africa region; Djibouti and Somalia from the Eastern sub-Saharan Africa region; and Cape Verde, Gambia, and Mauritania from the Western sub-Saharan Africa region. We also removed the island nation of São Tomé and Príncipe from the Western sub-Saharan Africa region despite having one year of survey data available because there was evidence that the traditional MC practices and prevalence was substantially different from the rest of the region.

After fitting each model, we generated 1,000 draws of all model parameters from the approximated joint posterior distribution using the inla.posterior.sample() function in R-INLA. For each draw *s* of the model parameters we constructed a draw of $p_{i,t}$ as:

$${p_{i,t}^{(s)}= logit}^{-1}\left( \beta_{0}^{(s)}+{\beta_{1}^{(s)}t+\gamma}_{c\left[ i \right]}^{(s)}+Z_{i,t}^{(s)}+ \epsilon_{i,t}^{(s)} \right)$$

Additional processing of the output from inla.posterior.sample() was required for the spatial-temporal random effect ($Z_{i,t}^{(s)}$) and the nugget effect ($\epsilon_{i,t}^{(s)}$) prior to constructing $p_{i,t}^{(s)}$ according to the equation above. We initially generated draws of $Z_{i,t}^{(s)}$ only at vertices of the finite element mesh, so we projected from this mesh to each combination of $\left( i, t \right)$ desired for prediction, encompassing the centroid of each grid cell on a 5 × 5-km grid as well as all years from 2000 to 2017. For the nugget effect, we generated $\epsilon_{i,t}^{(s)}$ for each combination of $\left( i, t \right)$ by sampling from $\mathrm{Normal}\left( 0, {\sigma_{nugget}^{2}}^{(s)} \right)$. We constructed 1,000 draws of $p_{i,t}$ for each grid cell and year combination through this process.

## 3.2 Model validation

The technical descriptions of methods for model validation are consistent with those previously used in the geospatial modelling of HIV.^2^

#### 3.2.1 Validation strategy

We assessed model performance using five-fold cross-validation with respect to predicting MC prevalence using a similar framework to previous research on HIV.^2^ We split all survey data into five groups by randomly sorting a list of unique identifiers for each survey, calculating the cumulative effective sample size of the surveys contained in this list, and then dividing the list into five parts at the point where this cumulative sample size was closest to 20%, 40%, 60%, and 80% of the total. This results in five approximately equal groups in terms of the total effective sample size that contained entire surveys (i.e., all of the data points derived from each survey are contained exclusively within only one fold). We then fit our model five times, excluding each of the five groups of data in turn.

After fitting, the data withheld from each of the five model were matched with predictions from that model, and then these data-prediction pairs were compiled across all models, resulting in a complete dataset of out-of-sample predictions corresponding to all survey data included in the analysis. Because MC prevalence estimates based on single survey clusters are generally quite noisy due to very small sample sizes, they do not constitute a reliable ‘gold standard’ for evaluating the model predictions. To address this issue, we aggregated both the observed data and the corresponding out-of-sample predictions within countries and within first and second administrative level units, by calculating a weighted mean of each using the effective sample sizes as the weights. We then calculate the mean error (ME; a measure of bias) and the root-mean-square error (RMSE; a measure of total variance) as summary measures across all data-estimate pairs.

Additionally, we constructed 95% prediction intervals for each data-estimate pair from the 2.5^th^ and 97.5^th^ percentiles of 1,000 draws from a binomial distribution corresponding to each of the 1,000 posterior draws of MC prevalence with *p* equal to MC prevalence in a given posterior draw and *N* equal to the effective sample size for the data point. We calculated coverage as the percentage of data-estimate pairs where the data point was contained within this 95% prediction interval.

Finally, to complement the out-of-sample predictive validity metrics, we also calculated in-sample predictive validity metrics using the same process but matching each data point to predictions from a model fit using all data.

#### 3.2.2 Model performance

We used this validation strategy to assess model performance of the model described above; summary error measures are presented in Supplementary Table 5. Out-of-sample validation metrics for MC coverage indicate good model fit with mean error close to zero (0.5 percentage-points); root-mean-square error of 3.6, 6.1, and 8.7 percentage-points for spatial stratification at the country, first, and second administrative levels, respectively; and a 95% prediction interval coverage of 96.96%.

## 3.3 Post estimation

#### 3.3.1 Fractional allocation of grid cells

In order to generate estimates of MC prevalence for first and second administrative level units, we first needed to determine the number of grid cells or fractions of a grid cell contained in each administrative unit. In order to accomplish this preliminary step, we intersected each grid cell with the second administrative level unit shape file to determine what fraction of the area of each grid cell fell within each administrative unit. Since all second administrative level units nest within first administrative level units, which in turn nest within countries, this strategy assigned the cell fractions to an administrative area at each level of the administrative hierarchy. We assumed that population density within each cell was uniform, and for cells that were split across multiple units, we allocated the WorldPop population estimate in proportion to area. This process was carried out separately for each modelling region, so cells that cross international borders that are also regional borders were allocated in their entirety to the country that contained the areal majority of the grid cell.

Using this assignment of cells and cell fractions to the administrative hierarchy, we scaled the grid-cell-level WorldPop estimates for the 15–49 age group to match the corresponding GBD population estimates for each country and year.^12^ To do so, for each country and year, we defined a population raking factor as the ratio of the GBD population estimate to the sum of the WorldPop population estimates for all cells and fractional cells within the country, and then multiplied the WorldPop population estimates for all cells and fractional cells within the country by this raking factor.

#### 3.3.2 Aggregation to first and second administrative level units

In addition to estimates of MC prevalence on a 5 × 5-km grid, we constructed estimates of MC prevalence for first and second administrative level units. These estimates were derived by calculating population-weighted averages of MC prevalence for each grid cell or fraction of a grid cell within a given first or second administrative level unit. This was carried out for each of the 1,000 posterior draws at the grid-cell level, generating 1,000 posterior draws for each administrative unit. Final point estimates and uncertainty intervals for each administrative unit at each level of the administrative hierarchy were derived from the mean, 2.5^th^ percentile, and 97.5^th^ percentile of these draws, respectively.

#### 3.3.3 Calculating number of circumcised and uncircumcised men

We estimated the number of circumcised and uncircumcised men in each grid cell and year by combining MC prevalence and one minus MC prevalence, respectively, with the estimated population of men ages 15–49 in that grid cell. Specifically, for each cell and fractional cell, we multiplied the estimated population by each of the 1,000 prevalence draws to generate 1,000 draws for the number of circumcised men. We generated 1,000 draws for the number of uncircumcised men for each grid cell and fraction of a grid cell by multiplying the estimated population of men ages 15–49 by one minus each of the 1,000 draws of MC prevalence. Fractional cells were then recombined by summing the estimated number of circumcised or uncircumcised men for each draw within each cell. Final point estimates and uncertainty intervals for the number of circumcised and uncircumcised men were calculated as the mean, 2.5^th^ percentile, and 97.5^th^ percentile of these draws, respectively.

# References

1 Global Health Data Exchange. GHDx. http://ghdx.healthdata.org (accessed Aug 22, 2018).

2 Dwyer-Lindgren L, Cork MA, Sligar A, *et al.* Mapping HIV prevalence in sub-Saharan Africa between 2000 and 2017. *Nature* 2019; **570**: 189–93.

3 Wiegand H. Kish, L.: Survey Sampling. John Wiley & Sons, Inc., New York, London 1965, IX + 643 S., 31 Abb., 56 Tab., Preis 83 s. *Biom Z* 1968; **10**: 88–9.

4 King G, Tomz M, Wittenberg J. Making the most of statistical analyses: improving interpretation and presentation. *Am J Polit Sci* 2000; **44**: 341–355.

5 GADM database of global administrative areas. https://gadm.org/ (accessed May 6, 2018) (accessed May 6, 2018).

6 WorldPop Population Dataset. https://www.worldpop.org/project/categories?id=3 (accessed July 7, 2017) (accessed July 7, 2017).

7 Franco-Villoria M, Ventrucci M, Rue H. Bayesian varying coefficient models using PC priors. *ArXiv180602084 Stat* 2018; published online June 6. http://arxiv.org/abs/1806.02084 (accessed June 6, 2019).

8 Fuglstad G-A, Simpson D, Lindgren F, Rue H. Constructing priors that penalize the complexity of Gaussian random fields. *J Am Stat Assoc* 2019; **114**: 445–52.

9 Lindgren F, Rue H, Lindström J. An explicit link between Gaussian fields and Gaussian Markov random fields: the stochastic partial differential equation approach. *J R Stat Soc Ser B Stat Methodol* 2011; **73**: 423–98.

10 Rue H, Martino S, Chopin N. Approximate Bayesian inference for latent Gaussian models by using integrated nested Laplace approximations. *J R Stat Soc Ser B Stat Methodol* 2009; **71**: 319–92.

11 James SL, Abate D, Abate KH, *et al.* Global, regional, and national incidence, prevalence, and years lived with disability for 354 diseases and injuries for 195 countries and territories, 1990–2017: a systematic analysis for the Global Burden of Disease Study 2017. *Lancet* 2018; **392**: 1789–858.

12 Murray CJL, Callender CSKH, Kulikoff XR, *et al.* Population and fertility by age and sex for 195 countries and territories, 1950–2017: a systematic analysis for the Global Burden of Disease Study 2017. *The Lancet* 2018; **392**: 1995–2051.

13 Land Processes Distributed Active Archive Center. Combined MODIS 5.1 dataset. MCD12Q1 LP DAAC NASA Land Data Prod. Serv. https://lpdaac.usgs.gov/dataset_discovery/modis/modis_products_table/mcd12q1/ (accessed June 1, 2017).

# Supplementary Figures


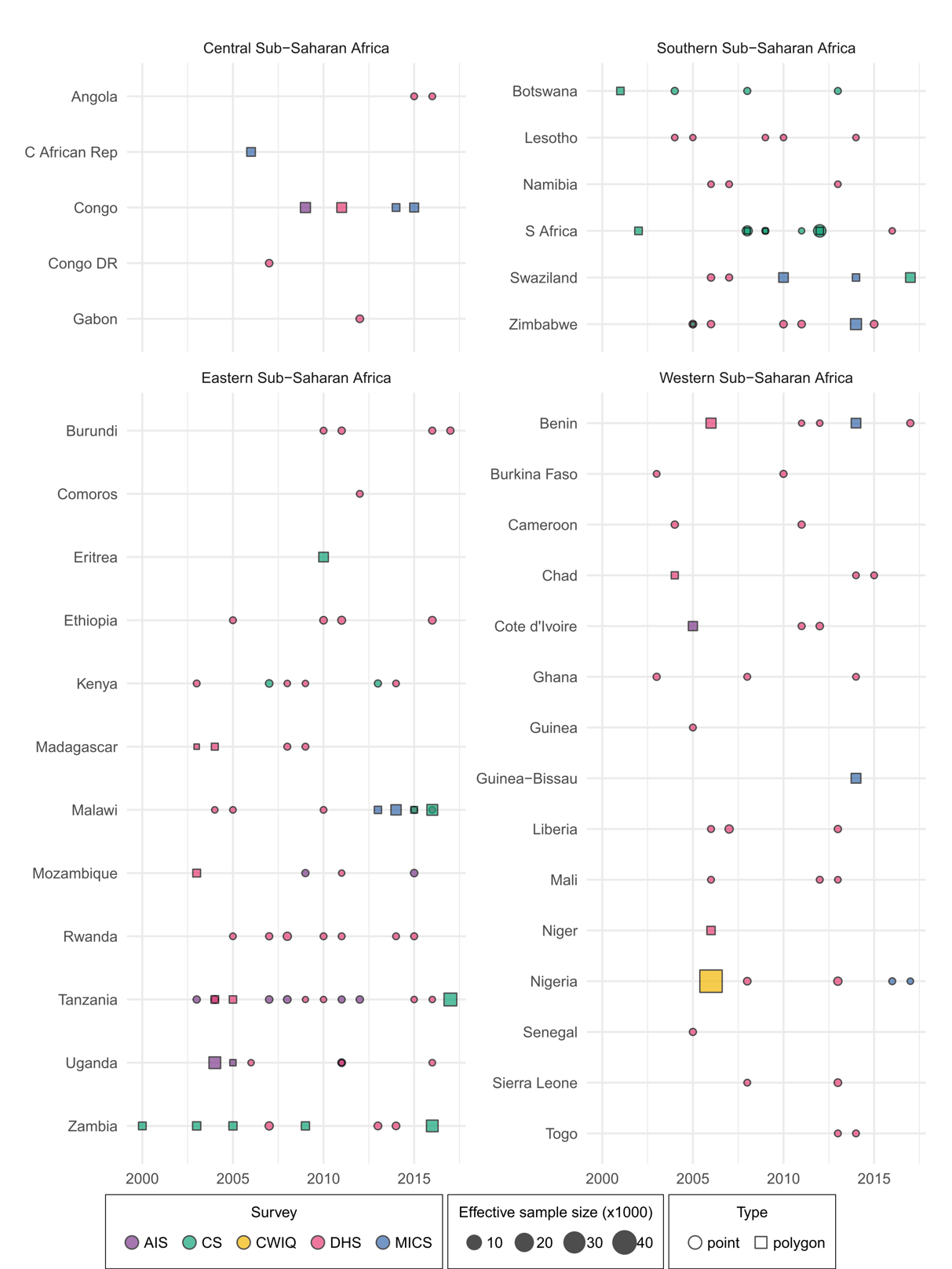


## Supplementary Figure 1: Male circumcision data availability.

MC survey data used in this analysis, by region and country. Size indicates the relative effective sample size for each source. Colour indicates the data source (AIS = AIDS Indicator Survey; CS = country-specific survey; CWIQ = Core Welfare Indicators Questionnaire Survey; DHS = Demographic and Health Survey; MICS = Multiple Indicator Cluster Survey). Shape type indicates whether a data source has point (GPS) or polygon location information. A full list of data sources with additional detail about data type (i.e., survey microdata or survey report) and geographical detail is given in Supplementary Table 1.


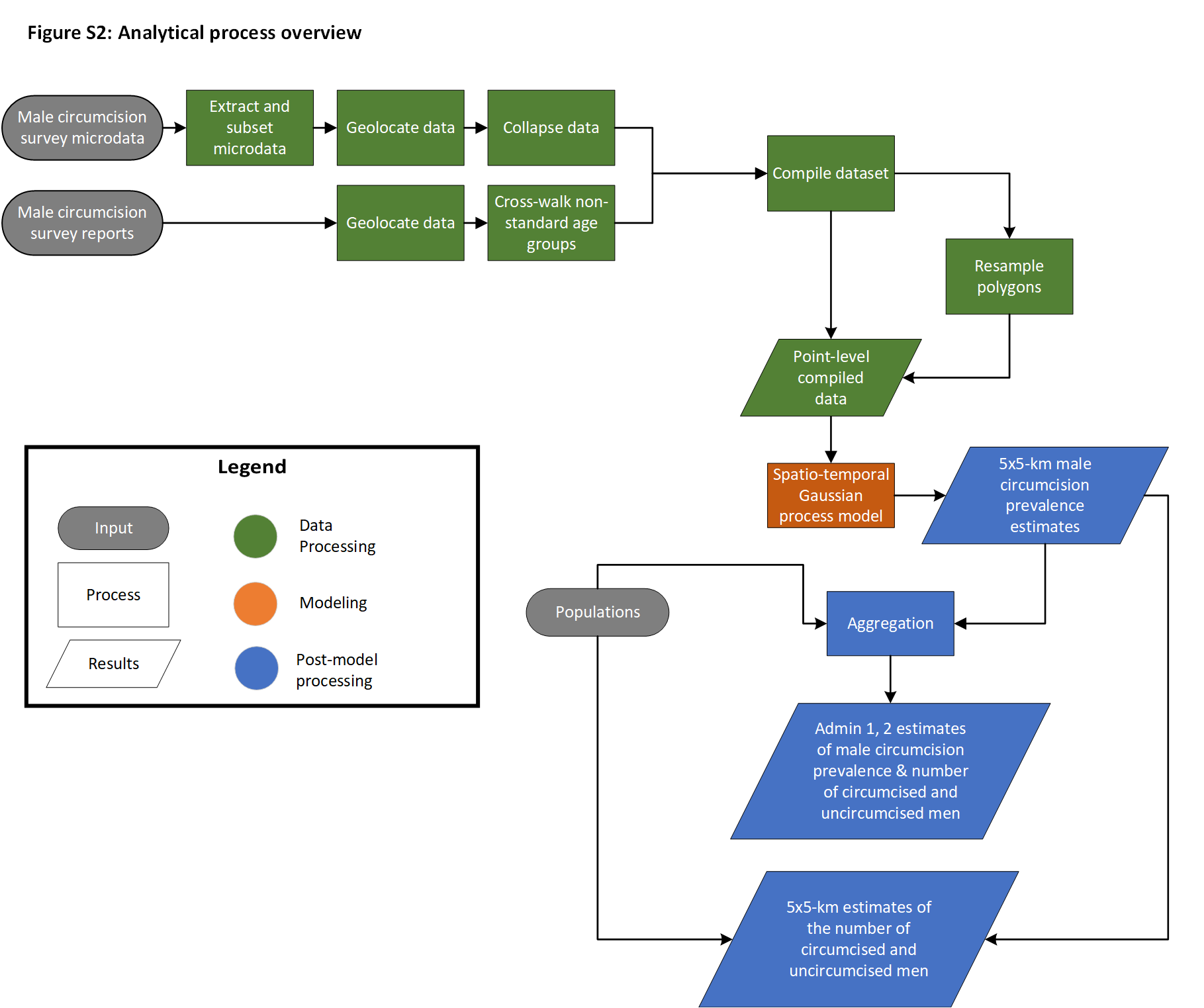


## Supplementary Figure 2: Analytic process overview.

The process used to produce MC prevalence estimates among males age 15–49 in sub-Saharan Africa involved three main parts. In the data processing steps (green), data were identified, extracted, and prepared for use in the MC prevalence model. In the modelling phase (orange), we use these data in a spatiotemporal Gaussian process model. In post-processing (blue), we aggregated prevalence estimates to the first and second administrative unit levels in each country and calculated the number of circumcised and circumcised men.


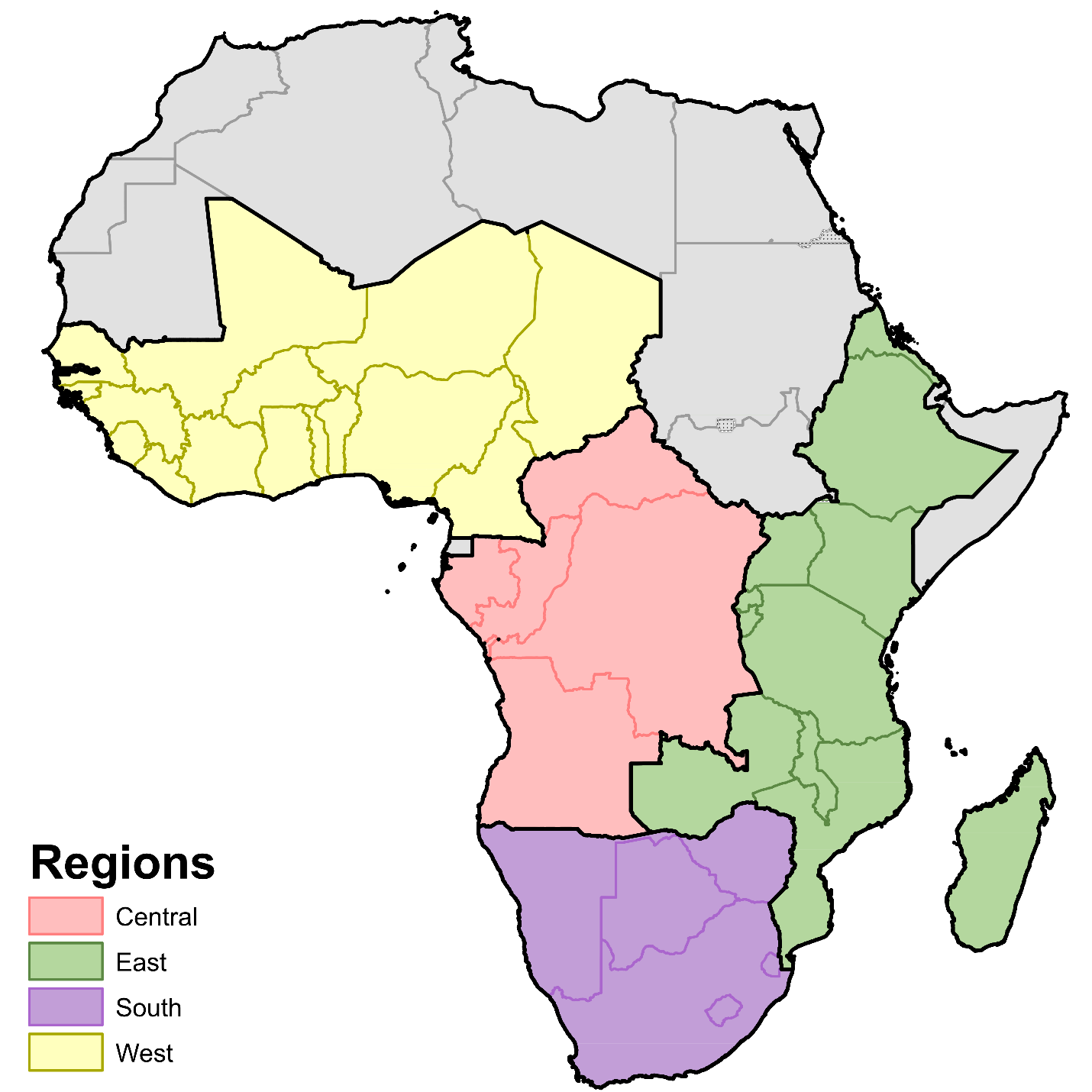


## Supplementary Figure 3: Modelling regions.

Modelling regions were based on the four Global Burden of Disease Study (GBD) regions in sub-Saharan Africa: Central, East, South, and West. We removed countries from our analysis if we did not find survey data during the years of our analysis. We also excluded the island nation of São Tomé and Príncipe despite having one year of survey data available because there was evidence that the traditional MC practices and prevalence was substantially different from the rest of the region. This resulted in several modifications to the GBD classification: we removed Equatorial Guinea from the Central sub-Saharan Africa region, Djibouti and Somalia from the Eastern sub-Saharan Africa region, and Cape Verde, Gambia, São Tomé and Príncipe, and Mauritania from the Western sub-Saharan Africa region. Countries in grey were not included in the analysis.


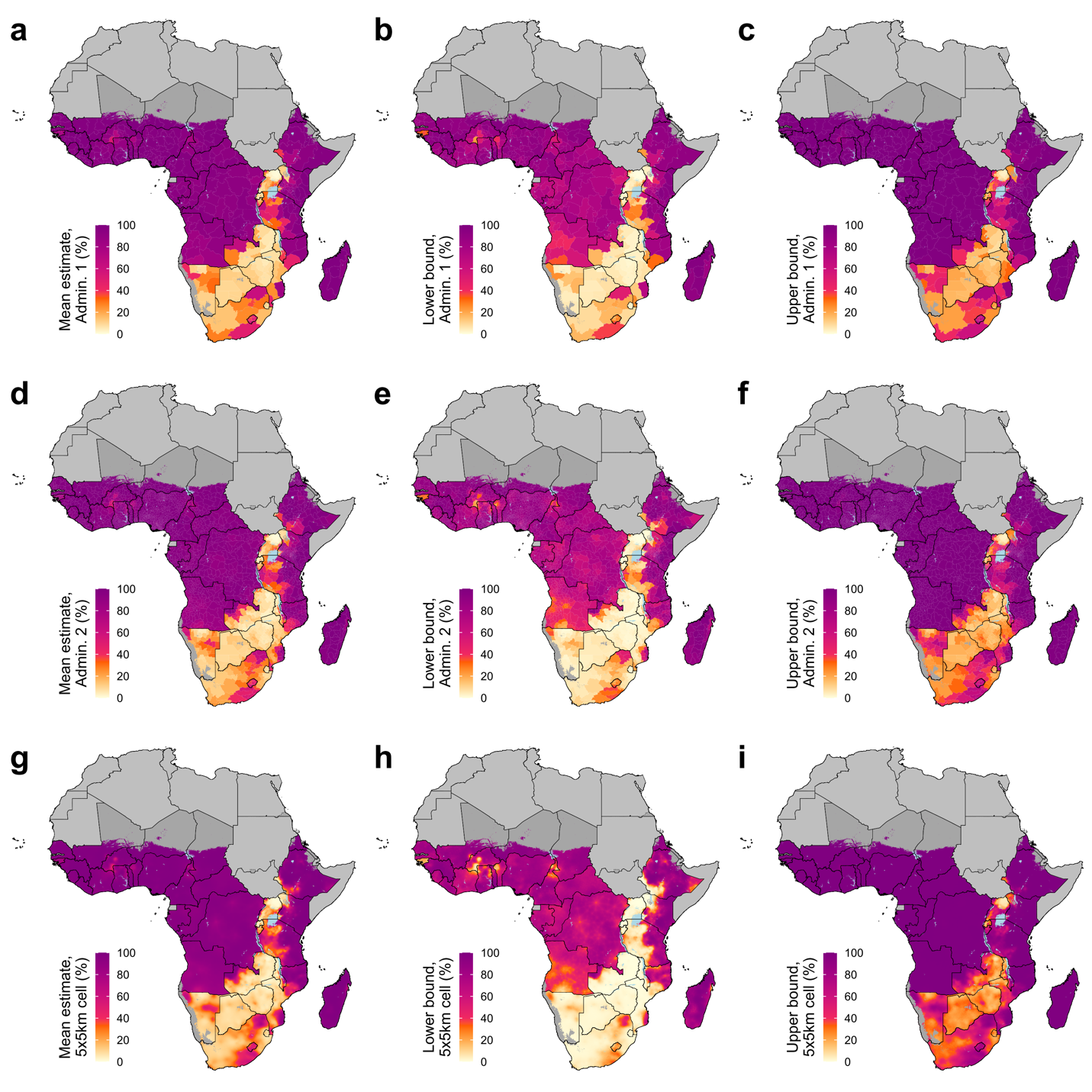


## Supplementary Figure 4: Estimated male circumcision prevalence, ages 15–49, 2000.

MC prevalence among males age 15–49 in 2000 at the first administrative unit level (**a–c**), second administrative unit level (**d–f**), and 5 × 5-km grid-cell level (**g–i**). Mean estimates and lower and upper bounds of the 95% uncertainty intervals are shown in the left, middle, and right columns, respectively. Maps reflect administrative boundaries, land cover, lakes, and population. Grid cells with fewer than ten people per 1 × 1-km^6^ and classified as ‘barren or sparsely vegetated’^13^ are colored in dark grey. Countries in light grey were not included in the analysis.


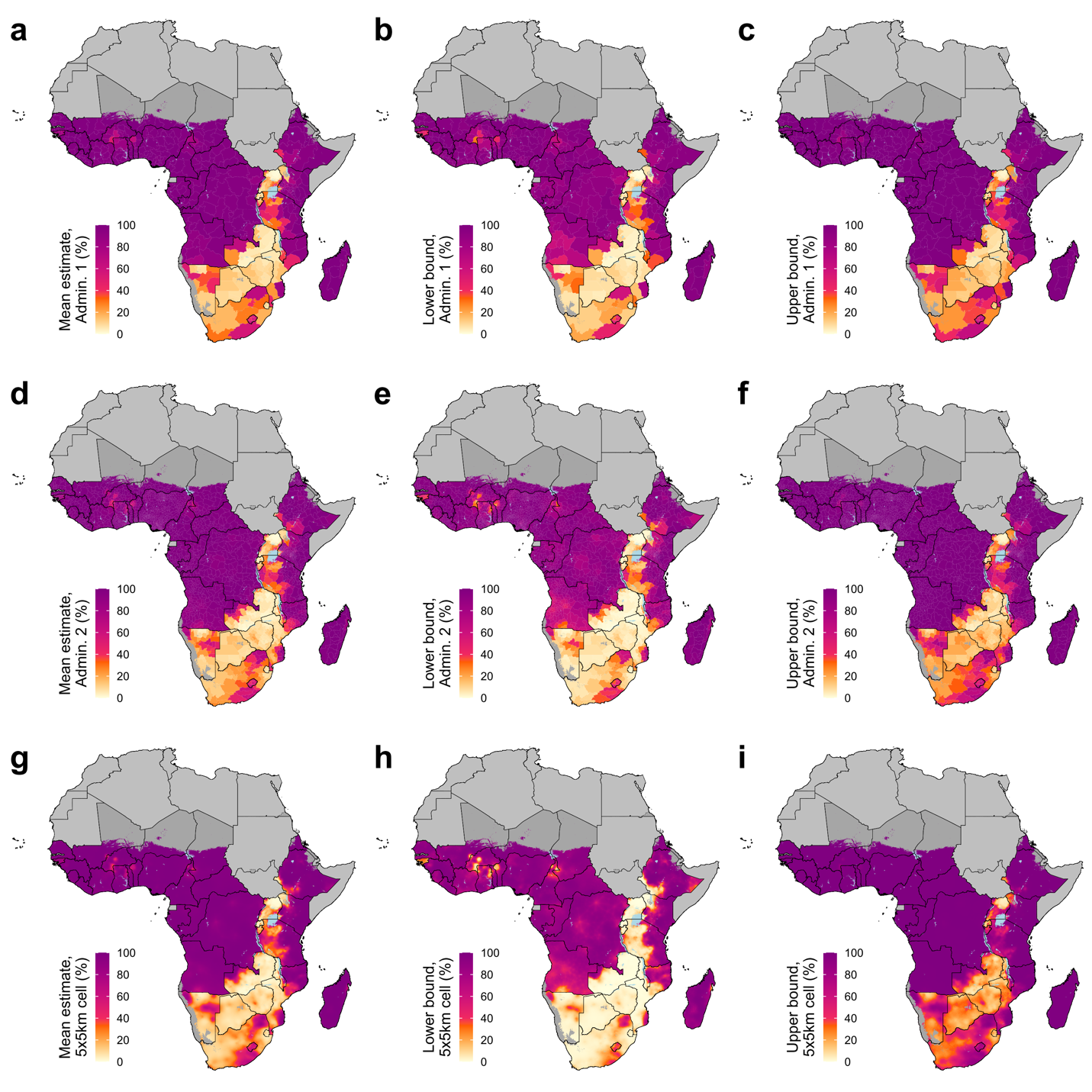


## Supplementary Figure 5: Estimated male circumcision prevalence, ages 15–49, 2005.

MC prevalence among males age 15–49 in 2005 at the first administrative unit level (**a–c**), second administrative unit level (**d–f**), and 5 × 5-km grid-cell level (**g–i**). Mean estimates and lower and upper bounds of the 95% uncertainty intervals are shown in the left, middle, and right columns, respectively. Maps reflect administrative boundaries, land cover, lakes, and population. Grid cells with fewer than ten people per 1 × 1-km^6^ and classified as ‘barren or sparsely vegetated’^13^ are colored in dark grey. Countries in light grey were not included in the analysis.


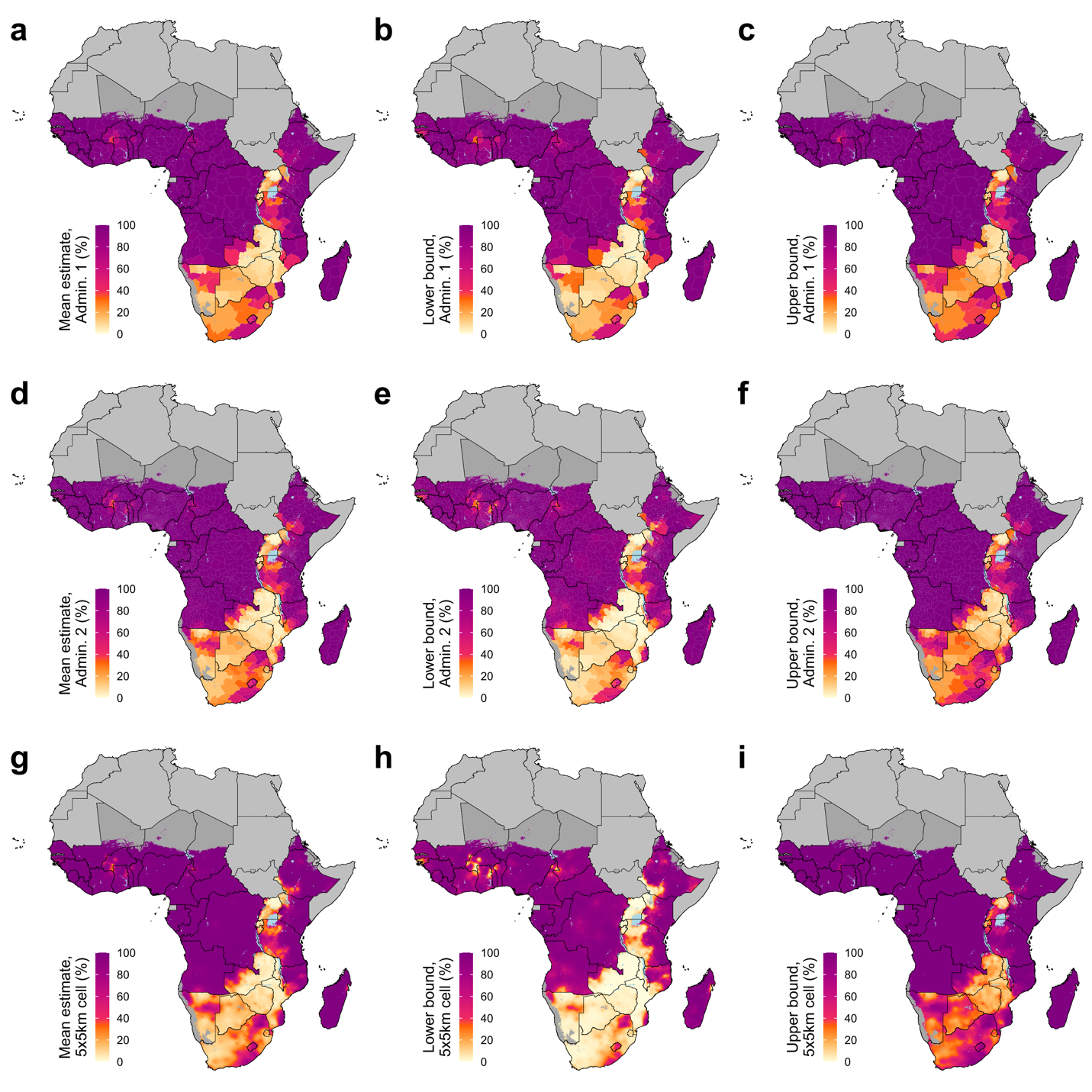


## Supplementary Figure 6: Estimated male circumcision prevalence, ages 15–49, 2010.

MC prevalence among males age 15–49 in 2010 at the first administrative unit level (**a–c**), second administrative unit level (**d–f**), and 5 × 5-km grid-cell level (**g–i**). Mean estimates and lower and upper bounds of the 95% uncertainty intervals are shown in the left, middle, and right columns, respectively. Maps reflect administrative boundaries, land cover, lakes, and population. Grid cells with fewer than ten people per 1 × 1-km^6^ and classified as ‘barren or sparsely vegetated’^13^ are colored in dark grey. Countries in light grey were not included in the analysis.


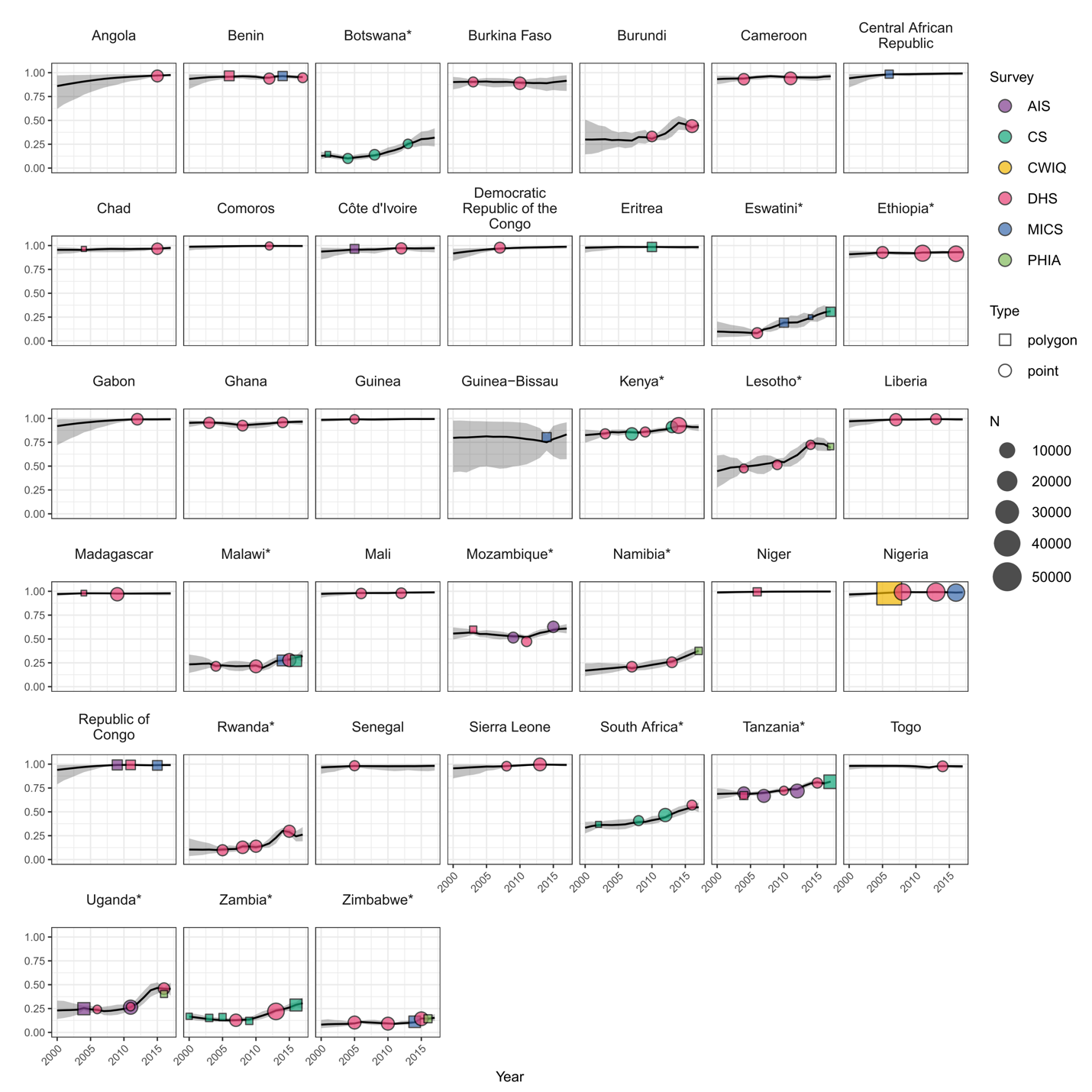


## Supplementary Figure 7: National data and estimates.

Data and estimates by country from 2000–2017. Estimates are displayed as a black line with uncertainty ranges in grey. Data are shown as points with colour indicating survey series (AIS = AIDS Indicator Survey; CS = Country-specific survey; DHS = Demographic and Health Survey; MICS = Multiple Indicator Cluster Survey; CWIQ = Core Welfare Indicators Questionnaire Survey), shape indicating the data type, and size indicating the effective sample size. Voluntary medical male circumcision (VMMC) priority countries, as identified by the WHO and UNAIDS, are indicated by an asterisk (*).


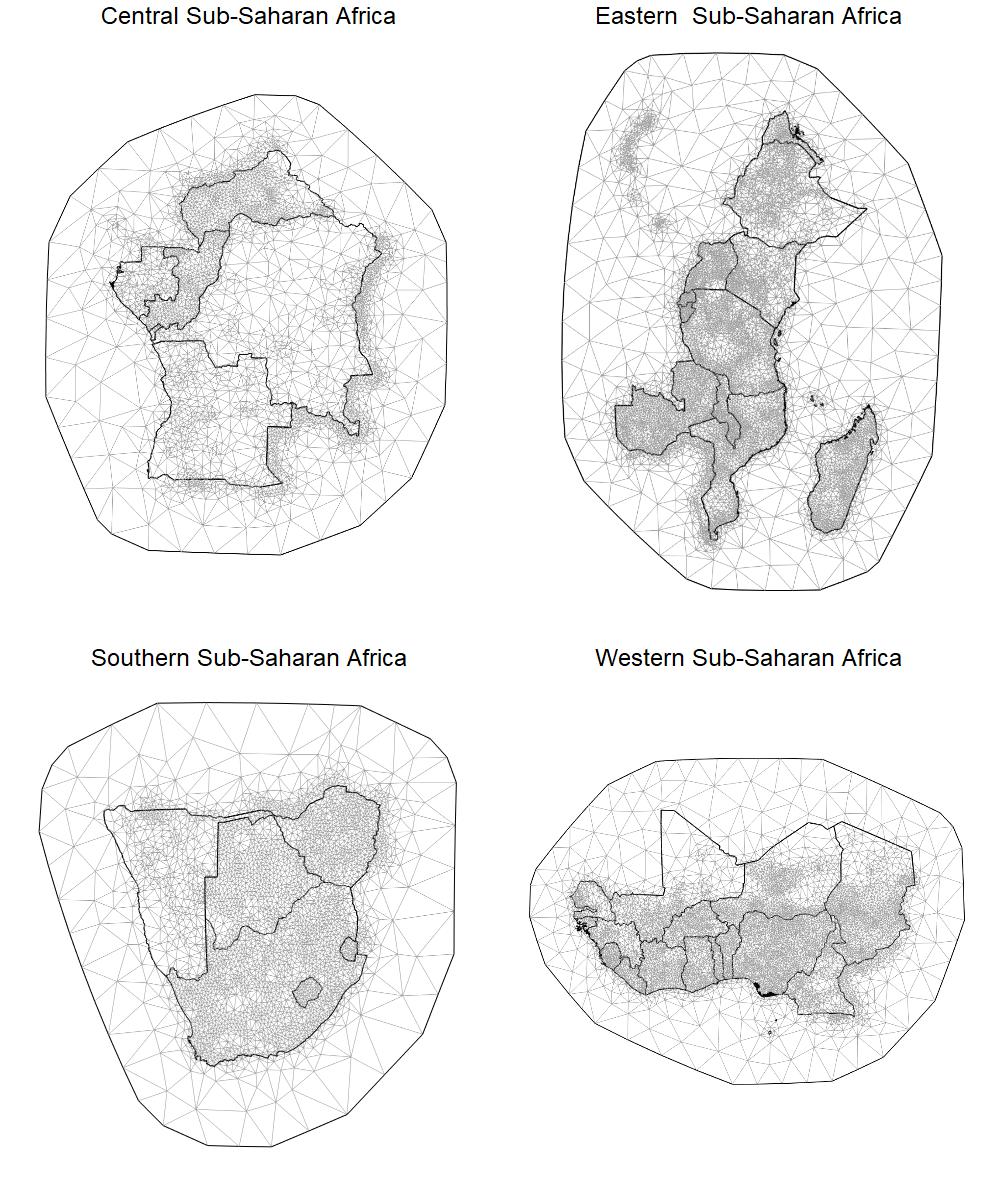


## Supplementary Figure 8: Space mesh for geostatistical models.

The finite elements mesh used to fit the space-time correlated random effect for each region overlaid on the countries in that region. Both the fine-scale mesh over land in the modelling region and the coarser buffer region mesh are shown.

# Supplementary Tables

## Supplementary Table 1: Male circumcision survey data.

*NID = Data source unique identifier in the Global Health Data Exchange.^1^ Additional information about each data sources is available *via* the GHDx, including information about the data provider and links to where the data can be accessed or requested (where available). NIDs can be entered in the search bar to retrieve the record for a particular source.

**Survey effective sample size for survey microdata was estimated *via* the Kish approximation, and for all survey reports the effective sample sizes was calculated by multiplying the median design effect (ratio of effective sample size to observed sample size) calculated in the microdata. For survey reports where MC estimates were for age groups other than 15–49 years, the cross-walk model calculated a new sample size that reflects our confidence in the estimate of MC prevalence as a function of the uncertainty in our linear model, original sample size, and the level of MC prevalence. Additional methodological details on calculating survey effective sample size are presented in appendix section 2.

| **Country** | **Year(s)** | **Name** | **Type** | **Geographical Detail** | **Citation** | **NID*** | **Survey sample size** | **Survey effective sample size**** |
| --- | --- | --- | --- | --- | --- | --- | --- | --- |
| Angola | 2015-16 | Angola Demographic and Health Survey 2015-2016 | Survey microdata | GPS | ICF International, Ministry of Health (Angola), National Institute of Statistics (Angola), United Nations Children's Fund (UNICEF). Angola Demographic and Health Survey 2015-2016. Fairfax, United States of America: ICF International, 2017. | 218555 | 5354 | 5354 |
| Benin | 2006 | Benin Demographic and Health Survey 2006 | Survey microdata | Admin 1 | Macro International, Inc, National Institute of Statistics and Economic Analysis (INSAE) (Benin), National Program Against AIDS (PNLS) (Benin). Benin Demographic and Health Survey 2006. Fairfax, United States of America: ICF International. | 18959 | 4604 | 4353 |
| Benin | 2011-12 | Benin Demographic and Health Survey 2011-2012 | Survey microdata | GPS | ICF International, National Institute of Statistics and Economic Analysis (INSAE) (Benin), National Program Against AIDS (PNLS) (Benin). Benin Demographic and Health Survey 2011-2012. Fairfax, United States of America: ICF International, 2014. | 79839 | 4363 | 4363 |
| Benin | 2014 | Benin Multiple Indicator Cluster Survey 2014 | Survey microdata | Admin 1 | National Institute of Statistics and Economic Analysis (INSAE) (Benin), United Nations Children's Fund (UNICEF). Benin Multiple Indicator Cluster Survey 2014. New York, United States of America: United Nations Children's Fund (UNICEF), 2017. | 206075 | 4370 | 3709 |
| Benin | 2017 | Benin Demographic and Health Survey 2017-2018 | Survey microdata | GPS | Hubert Koutoukou Maga National University Hospital Center (CNHU-HKM)(Benin), ICF International, National Institute of Statistics and Economic Analysis (INSAE) (Benin), National Malaria Control Program, Ministry of Health (Benin), Permanent Secretariat of the Food Council and Nutrition (SP-CAN)(Benin). Benin Demographic and Health Survey 2017-2018. Fairfax, United States of America: ICF International, 2018. | 218565 | 6537 | 3089 |
| Botswana | 2001 | Botswana AIDS Impact Survey 2001 | Survey microdata | Admin 2 | Central Statistics Office (Botswana), Ministry of Health (Botswana). Botswana AIDS Impact Survey 2001. Gaborone, Botswana: Statistics Botswana. | 22112 | 1323 | 1198 |
| Botswana | 2004 | Botswana AIDS Impact Survey 2004 | Survey microdata | GPS | Central Statistics Office (Botswana). Botswana AIDS Impact Survey 2004. Gaborone, Botswana: Statistics Botswana. | 22114 | 3346 | 3346 |
| Botswana | 2008 | Botswana AIDS Impact Survey 2008 | Survey microdata | GPS | Central Statistics Office (Botswana), National AIDS Coordinating Agency (Botswana). Botswana AIDS Impact Survey 2008. Gaborone, Botswana: Statistics Botswana, 2015. | 22116 | 3866 | 3865 |
| Botswana | 2013 | Botswana AIDS Impact Survey 2013 | Survey microdata | GPS | Ministry of Health (Botswana), National AIDS Coordinating Agency (Botswana), Statistics Botswana. Botswana AIDS Impact Survey 2013. Gaborone, Botswana: Statistics Botswana, 2015. | 134753 | 2763 | 2763 |
| Burkina Faso | 2003 | Burkina Faso Demographic and Health Survey 2003 | Survey microdata | GPS | Macro International, Inc, National Institute of Statistics and Demography (Burkina Faso). Burkina Faso Demographic and Health Survey 2003. Fairfax, United States of America: ICF International. | 19088 | 3167 | 3167 |
| Burkina Faso | 2010 | Burkina Faso Demographic and Health Survey 2010-2011 | Survey microdata | GPS | ICF Macro, Ministry of Health (Burkina Faso), National Institute of Statistics and Demography (Burkina Faso). Burkina Faso Demographic and Health Survey 2010-2011. Fairfax, United States of America: ICF International. | 19133 | 6144 | 6144 |
| Burundi | 2010-11 | Burundi Demographic and Health Survey 2010-2011 | Survey microdata | GPS | Burundi Institute of Statistics and Economic Studies, ICF International, Ministry of Public Health and the Fight Against AIDS (Burundi). Burundi Demographic and Health Survey 2010-2011. Fairfax, United States of America: ICF International, 2012. | 30431 | 3815 | 3815 |
| Burundi | 2016-17 | Burundi Demographic and Health Survey 2016-2017 | Survey microdata | GPS | Burundi Institute of Statistics and Economic Studies, ICF International, Ministry of Public Health and the Fight Against AIDS (Burundi). Burundi Demographic and Health Survey 2016-2017. Fairfax, United States of America: ICF International, 2018. | 286766 | 6680 | 6680 |
| Cameroon | 2004 | Cameroon Demographic and Health Survey 2004 | Survey microdata | GPS | Macro International, Inc, National Institute of Statistics (Cameroon). Cameroon Demographic and Health Survey 2004. Fairfax, United States of America: ICF International. | 19211 | 4770 | 4770 |
| Cameroon | 2011 | Cameroon Demographic and Health Survey 2011 | Survey microdata | GPS | ICF International, Ministry of Economy, Planning and Regional Development (Cameroon), Ministry of Public Health (Cameroon), National Institute of Statistics (Cameroon), Pasteur Center of Cameroon. Cameroon Demographic and Health Survey 2011. Fairfax, United States of America: ICF International. | 19274 | 6426 | 6426 |
| Central African Republic | 2006 | Central African Republic Multiple Indicator Cluster Survey 2006 | Survey microdata | Admin 1 | United Nations Children's Fund (UNICEF). Central African Republic Multiple Indicator Cluster Survey 2006. New York, United States: United Nations Children's Fund (UNICEF). | 2223 | 3530 | 2602 |
| Chad | 2004 | Chad Demographic and Health Survey 2004 | Survey microdata | Admin 1 | Macro International, Inc, National Institute for Statistics, Economic and Demographic Studies (INSEED) (Chad). Chad Demographic and Health Survey 2004. Fairfax, United States of America: ICF International. | 19315 | 1457 | 1069 |
| Chad | 2014-15 | Chad Demographic and Health Survey 2014-2015 | Survey microdata | GPS | ICF International, National Institute for Statistics, Economic and Demographic Studies (INSEED) (Chad). Chad Demographic and Health Survey 2014-2015. Fairfax, United States of America: ICF International, 2016. | 157025 | 4685 | 4685 |
| Comoros | 2012 | Comoros Demographic and Health Survey 2012-2013 | Survey microdata | GPS | General Directorate of Statistics and Forecasting (Comoros), ICF International. Comoros Demographic and Health Survey 2012-2013. Fairfax, United States of America: ICF International. | 76850 | 1901 | 1901 |
| Congo | 2009 | Congo AIDS Indicator Survey 2009 | Survey microdata | Admin 1 | ICF Macro, National Center for Statistics and Economic Studies (Congo, Rep.). Congo AIDS Indicator Survey 2009. Fairfax, United States of America: ICF International. | 3133 | 5856 | 4699 |
| Congo | 2011 | Congo Demographic and Health Survey 2011-2012 | Survey microdata | Admin 1 | ICF International, Ministry of Health (Congo, Rep.), National Center for Statistics and Economic Studies (Congo, Rep.). Congo Demographic and Health Survey 2011-2012. Fairfax, United States of America: ICF International. | 56151 | 4660 | 4063 |
| Congo | 2014-15 | Congo Multiple Indicator Cluster Survey 2014-2015 | Survey microdata | Admin 1 | National Institute of Statistics (INS) (Congo, Rep.), United Nations Children's Fund (UNICEF). Congo Multiple Indicator Cluster Survey 2014-2015. New York, United States of America: United Nations Children's Fund (UNICEF), 2018. | 234733 | 5088 | 4186 |
| Côte d'Ivoire | 2005 | Côte d'Ivoire AIDS Indicator Survey 2005 | Survey microdata | Admin >1 | CDC Retro-CI, Ministry of the Fight Against AIDS (Côte d'Ivoire), National Institute of Statistics (Côte d'Ivoire), ORC Macro. Côte d'Ivoire AIDS Indicator Survey 2005. Fairfax, United States of America: ICF International. | 56148 | 4481 | 3197 |
| Côte d'Ivoire | 2011-12 | Côte d'Ivoire Demographic and Health Survey 2011-2012 | Survey microdata | GPS | ICF International, Ministry of the Fight Against AIDS (Côte d'Ivoire), National Institute of Statistics (Côte d'Ivoire). Côte d'Ivoire Demographic and Health Survey 2011-2012. Fairfax, United States of America: ICF International. | 18533 | 4498 | 4498 |
| Democratic Republic of the Congo | 2007 | Democratic Republic of the Congo Demographic and Health Survey 2007 | Survey microdata | GPS | Macro International, Inc, Ministry of Planning (Congo, DR). Democratic Republic of the Congo Demographic and Health Survey 2007. Fairfax, United States of America: ICF International. | 19381 | 4220 | 4220 |
| Eritrea | 2010 | Eritrea Population and Health Survey 2010 | Survey report | Admin 1 | Kenya Medical Research Institute (KEMRI), National Statistics Office (Eritrea), The Fafo Research Foundation. Eritrea Population and Health Survey 2010. | 249999 | 4299 | 3783 |
| Eswatini | 2006-07 | Swaziland Demographic and Health Survey 2006-2007 | Survey microdata | GPS | Central Statistical Office (Swaziland), Macro International, Inc. Swaziland Demographic and Health Survey 2006-2007. Fairfax, United States of America: ICF International. | 20829 | 4085 | 4085 |
| Eswatini | 2010 | Swaziland Multiple Indicator Cluster Survey 2010 | Survey microdata | Admin 1 | Central Statistical Office (Swaziland), United Nations Children's Fund (UNICEF). Swaziland Multiple Indicator Cluster Survey 2010. New York, United States of America: United Nations Children's Fund (UNICEF). | 30325 | 3828 | 3559 |
| Eswatini | 2014 | Swaziland Multiple Indicator Cluster Survey 2014 | Survey microdata | Admin 1 | Central Statistical Office (Swaziland), United Nations Children's Fund (UNICEF), United Nations Educational, Scientific and Cultural Organization (UNESCO), United Nations Population Fund (UNFPA). Swaziland Multiple Indicator Cluster Survey 2014. New York, United States of America: United Nations Children's Fund (UNICEF), 2016. | 200707 | 1322 | 1039 |
| Eswatini | 2017 | Swaziland HIV Incidence Measurement Survey 2 2016-2017 | Survey microdata | Admin 1 | Centers for Disease Control and Prevention (CDC), Central Statistical Office (Swaziland), Environmental Health Department, Ministry of Health (Swaziland), Health Promotion Unit (Swaziland), ICAP, Columbia University Mailman School of Public Health, Ministry of Health (Swaziland), National Emergency Response Council on HIV and AIDS (NERCHA) (Swaziland), Swaziland Health Laboratory Services, Ministry of Health (SHLS), Swaziland Health Research (SHR), Swaziland National AIDS Programme (SNAP). Swaziland HIV Incidence Measurement Survey 2 2016-2017. | 415531 | 4042 | 3667 |
| Ethiopia | 2005 | Ethiopia Demographic and Health Survey 2005 | Survey microdata | GPS | Macro International, Inc, Population and Housing Census Commissions Office (PHCCO). Ethiopia Demographic and Health Survey 2005. Fairfax, United States of America: ICF International. | 19557 | 5387 | 5387 |
| Ethiopia | 2010-11 | Ethiopia Demographic and Health Survey 2010-2011 | Survey microdata | GPS | Central Statistical Agency (Ethiopia), ICF Macro, Ministry of Health (Ethiopia). Ethiopia Demographic and Health Survey 2010-2011. Fairfax, United States of America: ICF International. | 21301 | 12307 | 12307 |
| Ethiopia | 2016 | Ethiopia Demographic and Health Survey 2016 | Survey microdata | GPS | Central Statistical Agency (Ethiopia), ICF International. Ethiopia Demographic and Health Survey 2016. Fairfax, United States of America: ICF International, 2017. | 218568 | 11247 | 11247 |
| Gabon | 2012 | Gabon Demographic and Health Survey 2012 | Survey microdata | GPS | General Directorate of Statistics (Gabon), ICF International, Ministry of Economy, Employment and Sustainable Development (Gabon), Ministry of Health (Gabon). Gabon Demographic and Health Survey 2012. Fairfax, United States of America: ICF International, 2013. | 76706 | 4928 | 4928 |
| Ghana | 2003 | Ghana Demographic and Health Survey 2003 | Survey microdata | GPS | Ghana Statistical Service, Macro International, Inc. Ghana Demographic and Health Survey 2003. Fairfax, United States of America: ICF International. | 19627 | 4490 | 4490 |
| Ghana | 2008 | Ghana Demographic and Health Survey 2008 | Survey microdata | GPS | Ghana Statistical Service, Macro International, Inc, Ministry of Health (Ghana). Ghana Demographic and Health Survey 2008. Fairfax, United States of America: ICF International. | 21188 | 3935 | 3935 |
| Ghana | 2014 | Ghana Demographic and Health Survey 2014 | Survey microdata | GPS | Ghana Health Service, Ghana Statistical Service, ICF International. Ghana Demographic and Health Survey 2014. Fairfax, United States of America: ICF International, 2016. | 157027 | 3812 | 3812 |
| Guinea | 2005 | Guinea Demographic and Health Survey 2005 | Survey microdata | GPS | Macro International, Inc, National Statistics Directorate (Guinea). Guinea Demographic and Health Survey 2005. Fairfax, United States of America: ICF International. | 19683 | 2649 | 2649 |
| Guinea-Bissau | 2014 | Guinea-Bissau Multiple Indicator Cluster Survey 2014 | Survey microdata | Admin 1 | National Statistics Institute (Guinea-Bissau), United Nations Children's Fund (UNICEF). Guinea-Bissau Multiple Indicator Cluster Survey 2014. New York, United States of America: United Nations Children's Fund (UNICEF), 2016. | 174049 | 4232 | 3951 |
| Kenya | 2003 | Kenya Demographic and Health Survey 2003 | Survey microdata | GPS | Centers for Disease Control and Prevention (CDC), Central Bureau of Statistics (Kenya), Macro International, Inc, Ministry of Health (Kenya), National Council for Population and Development (Kenya). Kenya Demographic and Health Survey 2003. Fairfax, United States of America: ICF International. | 20145 | 3333 | 3333 |
| Kenya | 2007 | Kenya AIDS Indicator Survey 2007 | Survey microdata | GPS | Centers for Disease Control and Prevention (CDC), Kenya Medical Research Institute (KEMRI), Kenya National Bureau of Statistics, Ministry of Public Health and Sanitation (Kenya), National AIDS Control Council (Kenya), National AIDS and STI Control Programme (NASCOP) (Kenya), National Coordinating Agency for Population and Development (Kenya), National Public Health Laboratory Services, Ministry of Public Health and Sanitation (Kenya), United States Agency for International Development (USAID). Kenya AIDS Indicator Survey 2007. Nairobi, Kenya: Kenya National Bureau of Statistics. | 133219 | 6193 | 6193 |
| Kenya | 2008-09 | Kenya Demographic and Health Survey 2008-2009 | Survey microdata | GPS | ICF Macro, Kenya Medical Research Institute (KEMRI), Kenya National Bureau of Statistics, Ministry of Public Health and Sanitation (Kenya), National AIDS and STI Control Programme (NASCOP) (Kenya), National Aids Control Council (NACC), National Coordinating Agency for Population and Development (Kenya). Kenya Demographic and Health Survey 2008-2009. Fairfax, United States of America: ICF International. | 21365 | 3246 | 3246 |
| Kenya | 2013 | Kenya AIDS Indicator Survey 2012-2013 | Survey microdata | GPS | Kenya National Bureau of Statistics, Ministry of Devolution and Planning (Kenya), Ministry of Health (Kenya), National AIDS and STI Control Programme (NASCOP) (Kenya). Kenya AIDS Indicator Survey 2012-2013. Nairobi, Kenya: Kenya National Bureau of Statistics. | 133304 | 4874 | 4874 |
| Kenya | 2014 | Kenya Demographic and Health Survey 2014 | Survey microdata | GPS | ICF International, Kenya Medical Research Institute (KEMRI), Kenya National Bureau of Statistics, Ministry of Health (Kenya), National AIDS Control Council (Kenya), National Council for Population and Development (Kenya). Kenya Demographic and Health Survey 2014. Fairfax, United States of America: ICF International. | 157057 | 11924 | 11924 |
| Lesotho | 2004-05 | Lesotho Demographic and Health Survey 2004-2005 | Survey microdata | GPS | Bureau of Statistics (Lesotho), Macro International, Inc, Ministry of Health and Social Welfare (Lesotho). Lesotho Demographic and Health Survey 2004-2005. Fairfax, United States of America: ICF International. | 20167 | 2353 | 2353 |
| Lesotho | 2009-10 | Lesotho Demographic and Health Survey 2009-2010 | Survey microdata | GPS | ICF Macro, Ministry of Health and Social Welfare (Lesotho). Lesotho Demographic and Health Survey 2009-2010. Fairfax, United States of America: ICF International. | 21382 | 2952 | 2952 |
| Lesotho | 2014 | Lesotho Demographic and Health Survey 2014 | Survey microdata | GPS | ICF International, Ministry of Health and Social Welfare (Lesotho). Lesotho Demographic and Health Survey 2014. Fairfax, United States of America: ICF International. | 157058 | 2624 | 2624 |
| Lesotho | 2017 | Lesotho Population-based HIV Impact Assessment 2016-2017 | Survey report | Admin 1 | Bureau of Statistics (Lesotho), Centers for Disease Control and Prevention (CDC), ICAP, Columbia University Mailman School of Public Health, Ministry of Health (Lesotho). Lesotho Population-Based HIV Impact Assessment 2016-2017. | 327582 | 5361 | 1441 |
| Liberia | 2006-07 | Liberia Demographic and Health Survey 2006-2007 | Survey microdata | GPS | Liberia Institute for Statistics and Geo-information Services (LISGIS), Macro International, Inc. Liberia Demographic and Health Survey 2006-2007. Fairfax, United States of America: ICF International. | 20191 | 5831 | 5826 |
| Liberia | 2013 | Liberia Demographic and Health Survey 2013 | Survey microdata | GPS | ICF International, Liberia Institute for Statistics and Geo-information Services (LISGIS), National AIDS and STI Control Program (NACP), Ministry of Health and Social Welfare (Liberia). Liberia Demographic and Health Survey 2013. Fairfax, United States of America: ICF International. | 77385 | 4111 | 4111 |
| Madagascar | 2003-04 | Madagascar Demographic and Health Survey 2003-2004 | Survey microdata | Admin 2 | Macro International, Inc, National Institute of Statistics (Madagascar). Madagascar Demographic and Health Survey 2003-2004. Fairfax, United States of America: ICF International. | 20223 | 2200 | 1175 |
| Madagascar | 2008-09 | Madagascar Demographic and Health Survey 2008-2009 | Survey microdata | GPS | ICF Macro, National Institute of Statistics (Madagascar). Madagascar Demographic and Health Survey 2008-2009. Fairfax, United States of America: ICF International. | 21409 | 7341 | 7341 |
| Malawi | 2004-05 | Malawi Demographic and Health Survey 2004-2005 | Survey microdata | GPS | Macro International, Inc, National Statistical Office of Malawi. Malawi Demographic and Health Survey 2004-2005. Fairfax, United States of America: ICF International. | 20263 | 3001 | 3001 |
| Malawi | 2010 | Malawi Demographic and Health Survey 2010 | Survey microdata | GPS | ICF Macro, National Statistical Office of Malawi. Malawi Demographic and Health Survey 2010. Fairfax, United States of America: ICF International. | 21393 | 6637 | 6637 |
| Malawi | 2013-14 | Malawi Multiple Indicator Cluster Survey 2013-2014 | Survey microdata | Admin 2 | National Statistical Office of Malawi, United Nations Children's Fund (UNICEF). Malawi Multiple Indicator Cluster Survey 2013-2014. New York, United States of America: United Nations Children's Fund (UNICEF), 2015. | 161662 | 6842 | 5733 |
| Malawi | 2015-16 | Malawi Demographic and Health Survey 2015-2016 | Survey microdata | GPS | Emory University and Centers for Disease Control & Prevention Collaboration, ICF International, Ministry of Health (Malawi), National Statistical Office of Malawi. Malawi Demographic and Health Survey 2015-2016. Fairfax, United States of America: ICF International, 2017. | 218581 | 7128 | 7128 |
| Malawi | 2015-16 | Malawi Population-based HIV Impact Assessment 2015-2016 | Survey microdata | Admin >1 | Ministry of Health, Malawi. Malawi Population-based HIV Impact Assessment (MPHIA) 2015-16: First Report. Lilongwe, Ministry of Health. November 2017. http://phia.icap.columbia.edu | 287629 | 7076 | 6563 |
| Mali | 2006 | Mali Demographic and Health Survey 2006 | Survey microdata | GPS | Macro International, Inc, Ministry of Health (Mali), National Directorate of Statistics and Informatics (DNSI) (Mali). Mali Demographic and Health Survey 2006. Fairfax, United States of America: ICF International. | 20274 | 3672 | 3672 |
| Mali | 2012-13 | Mali Demographic and Health Survey 2012-2013 | Survey microdata | GPS | ICF International, INFO-STAT (Mali), Ministry of Health (Mali), National Institute of Statistics (INSTAT) (Mali), Planning and Statistics Unit, Ministry of Health (Mali). Mali Demographic and Health Survey 2012-2013. Fairfax, United States of America: ICF International, 2014. | 77388 | 3810 | 3810 |
| Mozambique | 2003 | Mozambique Demographic and Health Survey 2003-2004 | Survey microdata | Admin 1 | Macro International, Inc, National Institute of Statistics (INE) (Mozambique). Mozambique Demographic and Health Survey 2003-2004. Fairfax, United States of America: ICF International. | 20394 | 2495 | 1867 |
| Mozambique | 2009 | Mozambique AIDS Indicator Survey 2009 | Survey microdata | GPS | ICF Macro, Ministry of Health (Mozambique), National Institute of Statistics (INE) (Mozambique). Mozambique AIDS Indicator Survey 2009. Fairfax, United States of America: ICF International, 2010. | 8906 | 4145 | 4145 |
| Mozambique | 2011 | Mozambique Demographic and Health Survey 2011 | Survey microdata | GPS | ICF Macro, Manhica Health Research Center (CISM), Ministry of Health (Mozambique), National Institute of Statistics (INE) (Mozambique). Mozambique Demographic and Health Survey 2011. Fairfax, United States of America: ICF International. | 55975 | 3508 | 3508 |
| Mozambique | 2015 | Mozambique AIDS Indicator Survey 2015 | Survey microdata | GPS | Centers for Disease Control and Prevention (CDC), ICF International, Ministry of Health (Mozambique), National Institute of Health (Mozambique), National Institute of Statistics (INE) (Mozambique). Mozambique AIDS Indicator Survey 2015. Fairfax, United States of America: ICF International, 2018. | 157060 | 4726 | 4726 |
| Namibia | 2006-07 | Namibia Demographic and Health Survey 2006-2007 | Survey microdata | GPS | Macro International, Inc, Ministry of Health and Social Services (Namibia). Namibia Demographic and Health Survey 2006-2007. Fairfax, United States of America: ICF International. | 20428 | 3848 | 3848 |
| Namibia | 2013 | Namibia Demographic and Health Survey 2013 | Survey microdata | GPS | ICF International, Ministry of Health and Social Services (Namibia), Namibia Institute of Pathology, Namibia Statistics Agency. Namibia Demographic and Health Survey 2013. Fairfax, United States of America: ICF International. | 150382 | 3919 | 3919 |
| Namibia | 2017 | Namibia Population-based HIV Impact Assessment 2017 | Survey report | Admin 1 | Centers for Disease Control and Prevention (CDC), ICAP, Columbia University Mailman School of Public Health, Ministry of Health and Social Services (Namibia), Namibia Institute of Pathology, Namibia Statistics Agency, University of California San Francisco. Namibia Population-Based HIV Impact Assessment 2017. | 359089 | 8271 | 2003 |
| Niger | 2006 | Niger Demographic and Health Survey 2006 | Survey microdata | Admin 1 | Department of Statistics and National Accounts (Niger), Macro International, Inc. Niger Demographic and Health Survey 2006. Fairfax, United States of America: ICF International. | 20499 | 3117 | 2632 |
| Nigeria | 2006 | Nigeria Core Welfare Indicators Questionnaire Survey 2006 | Survey microdata | Admin 1 | National Bureau of Statistics (Nigeria). Nigeria Core Welfare Indicators Questionnaire Survey 2006. Abuja, Nigeria: National Bureau of Statistics (Nigeria). | 9522 | 85149 | 51037 |
| Nigeria | 2008 | Nigeria Demographic and Health Survey 2008 | Survey microdata | GPS | Macro International, Inc, National Population Commission of Nigeria. Nigeria Demographic and Health Survey 2008. Fairfax, United States of America: ICF International, 2009. | 21433 | 13666 | 13666 |
| Nigeria | 2013 | Nigeria Demographic and Health Survey 2013 | Survey microdata | GPS | ICF International, National Population Commission of Nigeria. Nigeria Demographic and Health Survey 2013. Fairfax, United States of America: ICF International. | 77390 | 17170 | 17170 |
| Nigeria | 2016-17 | Nigeria Multiple Indicator Cluster Survey with National Immunization Coverage Survey Supplement 2016-2017 | Survey microdata | GPS | National Agency for the Control of AIDS (Nigeria), National Bureau of Statistics (Nigeria), National Primary Health Care Development Agency (NPHCDA) (Nigeria), United Nations Children's Fund (UNICEF). Nigeria Multiple Indicator Cluster Survey with National Immunization Coverage Survey Supplement 2016-2017. New York, United States of America: United Nations Children's Fund (UNICEF), 2018. | 218613 | 15153 | 15153 |
| Rwanda | 2005 | Rwanda Demographic and Health Survey 2005 | Survey microdata | GPS | Macro International, Inc, National Institute of Statistics of Rwanda. Rwanda Demographic and Health Survey 2005. Fairfax, United States of America: ICF International. | 20740 | 4339 | 4339 |
| Rwanda | 2007-08 | Rwanda Interim Demographic and Health Survey 2007-2008 | Survey microdata | GPS | Macro International, Inc, Ministry of Health (Rwanda), National Institute of Statistics of Rwanda. Rwanda Interim Demographic and Health Survey 2007-2008. Fairfax, United States of America: ICF International. | 21222 | 6132 | 6132 |
| Rwanda | 2010-11 | Rwanda Demographic and Health Survey 2010-2011 | Survey microdata | GPS | ICF Macro, Ministry of Health (Rwanda), National Institute of Statistics of Rwanda. Rwanda Demographic and Health Survey 2010-2011. Fairfax, United States of America: ICF International. | 56040 | 5689 | 5689 |
| Rwanda | 2014-15 | Rwanda Demographic and Health Survey 2014-2015 | Survey microdata | GPS | ICF International, Ministry of Health (Rwanda), National Institute of Statistics of Rwanda. Rwanda Demographic and Health Survey 2014-2015. Fairfax, United States of America: ICF International, 2016. | 157063 | 5580 | 5580 |
| Senegal | 2005 | Senegal Demographic and Health Survey 2005 | Survey microdata | GPS | Ministry of Health and Prevention (Senegal), Research Center for Human Development (Senegal). Senegal Demographic and Health Survey 2005. Fairfax, United States of America: ICF International. | 26855 | 3322 | 3322 |
| Sierra Leone | 2008 | Sierra Leone Demographic and Health Survey 2008 | Survey microdata | GPS | Macro International, Inc, Statistics Sierra Leone. Sierra Leone Demographic and Health Survey 2008. Fairfax, United States of America: ICF International. | 21258 | 2885 | 2885 |
| Sierra Leone | 2013 | Sierra Leone Demographic and Health Survey 2013 | Survey microdata | GPS | ICF International, Ministry of Health and Sanitation (Sierra Leone), Statistics Sierra Leone. Sierra Leone Demographic and Health Survey 2013. Fairfax, United States of America: ICF International, 2014. | 131467 | 6563 | 6563 |
| South Africa | 2002 | South Africa HIV/AIDS Behavioral Risks, Sero-Status, and Mass Media Impact Survey 2002 | Survey microdata | Admin 3 | Center for AIDS Development, Research and Evaluation (CADRE) (South Africa), Department of Social Development (South Africa), Family Health International, Geospace International (South Africa), Human Sciences Research Council, Joint United Nations Program on HIV/AIDS (UNAIDS), Medical University of Southern Africa (MEDUNSA), National Agency for AIDS Research (ANRS) (France), National Health Laboratory Service (NHLS) (South Africa), South African Medical Research Council, University of Natal, Wits Health Consortium. South Africa HIV/AIDS Behavioral Risks, Sero-Status, and Mass Media Impact Survey 2002. Pretoria, South Africa: Human Sciences Research Council, 2011. | 12102 | 2422 | 1270 |
| South Africa | 2008-09 | South Africa National HIV Prevalence, Incidence, Behavior and Communication Survey 2008-2009 | Survey microdata | Admin 5, GPS | Center for AIDS Development, Research and Evaluation (CADRE) (South Africa), Geospace International (South Africa), Global Clinical and Viral Laboratory (South Africa), Human Sciences Research Council, Maphume Research Services, National Institute for Communicable Diseases (South Africa), South African Medical Research Council. South Africa National HIV Prevalence, Incidence, Behavior and Communication Survey 2008-2009. Pretoria, South Africa: Human Sciences Research Council, 2014. | 228102 | 3995 | 3346 |
| South Africa | 2011-12 | South Africa National HIV Prevalence, Incidence, and Behavior Survey 2011-2012 | Survey microdata | Admin 5, GPS | Centers for Disease Control and Prevention (CDC), Global Clinical and Viral Laboratory (South Africa), Human Sciences Research Council, National Institute for Communicable Diseases (South Africa), South African Medical Research Council, University of Cape Town. South Africa National HIV Prevalence, Incidence, and Behavior Survey 2011-2012. Pretoria, South Africa: Human Sciences Research Council, 2016. | 313076 | 8269 | 7303 |
| South Africa | 2016 | South Africa Demographic and Health Survey 2016 | Survey microdata | GPS | Department of Health (South Africa), ICF International, South African Medical Research Council, Statistics South Africa. South Africa Demographic and Health Survey 2016. Fairfax, United States of America: ICF International, 2019. | 157064 | 3172 | 3172 |
| Togo | 2013-14 | Togo Demographic and Health Survey 2013-2014 | Survey microdata | GPS | Directorate General of Statistics and National Accounts (Togo), ICF International, Ministry of Health (Togo), Ministry of Planning, Development and Zoning (Togo). Togo Demographic and Health Survey 2013-2014. Fairfax, United States of America: ICF International, 2015. | 77515 | 4006 | 4006 |
| Uganda | 2004-05 | Uganda AIDS Indicator Survey 2004-2005 | Survey microdata | Admin 1 | Division of Reproductive Health, Centers for Disease Control and Prevention (CDC), Ministry of Health (Uganda). Uganda AIDS Indicator Survey 2004-2005. | 13084 | 8001 | 8001 |
| Uganda | 2006 | Uganda Demographic and Health Survey 2006 | Survey microdata | GPS | Macro International, Inc, Uganda Bureau of Statistics. Uganda Demographic and Health Survey 2006. Fairfax, United States of America: ICF International. | 21014 | 2167 | 2167 |
| Uganda | 2011 | Uganda AIDS Indicator Survey 2011 | Survey microdata | GPS | Centers for Disease Control and Prevention (CDC), ICF Macro, Ministry of Health (Uganda), Uganda Bureau of Statistics, Uganda Virus Research Institute. Uganda AIDS Indicator Survey 2011. Fairfax, United States of America: ICF International. | 55973 | 8718 | 8718 |
| Uganda | 2011 | Uganda Demographic and Health Survey 2011 | Survey microdata | GPS | ICF Macro, Uganda Bureau of Statistics. Uganda Demographic and Health Survey 2011. Fairfax, United States of America: ICF International. | 56021 | 2175 | 2175 |
| Uganda | 2016 | Uganda Demographic and Health Survey 2016 | Survey microdata | GPS | ICF International, Uganda Bureau of Statistics. Uganda Demographic and Health Survey 2016. Fairfax, United States of America: ICF International, 2018. | 286780 | 4970 | 4970 |
| Uganda | 2016 | Uganda Population-based HIV Impact Assessment 2016-2017 | Survey report | Admin 1 | Centers for Disease Control and Prevention (CDC), ICAP, Columbia University Mailman School of Public Health, Joint United Nations Program on HIV/AIDS (UNAIDS), Ministry of Health (Uganda), Uganda Bureau of Statistics, Uganda Virus Research Institute, World Health Organization (WHO). Uganda Population-Based HIV Impact Assessment 2016-2017. | 327593 | 12544 | 1780 |
| United Republic of Tanzania | 2003-04 | Tanzania AIDS Indicator Survey 2003-2004 | Survey microdata | GPS | National Bureau of Statistics (Tanzania), ORC Macro, Tanzania Commission for AIDS (TACAIDS). Tanzania AIDS Indicator Survey 2003-2004. Fairfax, United States of America: ICF International. | 12630 | 5652 | 5652 |
| United Republic of Tanzania | 2004-05 | Tanzania Demographic and Health Survey 2004-2005 | Survey microdata | Admin 1 | Macro International, Inc, National Bureau of Statistics (Tanzania). Tanzania Demographic and Health Survey 2004-2005. Fairfax, United States of America: ICF International. | 20875 | 2634 | 2544 |
| United Republic of Tanzania | 2007-08 | Tanzania HIV/AIDS and Malaria Indicator Survey 2007-2008 | Survey microdata | GPS | Macro International, Inc, National Bureau of Statistics (Tanzania), Office of the Chief Government Statistician (OCGS) (Zanzibar), Tanzania Commission for AIDS (TACAIDS), Zanzibar AIDS Commission (ZAC). Tanzania HIV/AIDS and Malaria Indicator Survey 2007-2008. Fairfax, United States of America: ICF International. | 12644 | 6862 | 6862 |
| United Republic of Tanzania | 2009-10 | Tanzania Demographic and Health Survey 2009-2010 | Survey microdata | GPS | ICF Macro, National Bureau of Statistics (Tanzania). Tanzania Demographic and Health Survey 2009-2010. Fairfax, United States of America: ICF International. | 21331 | 2433 | 2433 |
| United Republic of Tanzania | 2011-12 | Tanzania AIDS Indicator Survey 2011-2012 | Survey microdata | GPS | ICF International, National Bureau of Statistics (Tanzania), Office of the Chief Government Statistician (OCGS) (Zanzibar), Tanzania Commission for AIDS (TACAIDS), Zanzibar AIDS Commission (ZAC). Tanzania AIDS Indicator Survey 2011-2012. Fairfax, United States of America: ICF International, 2013. | 77395 | 8179 | 8179 |
| United Republic of Tanzania | 2015-16 | Tanzania Demographic and Health Survey 2015-2016 | Survey microdata | GPS | ICF International, Ministry of Health (Zanzibar), Ministry of Health, Community Development, Gender, Elderly and Children (MoHCDEC) (Tanzania), National Bureau of Statistics (Tanzania), Office of the Chief Government Statistician (OCGS) (Zanzibar). Tanzania Demographic and Health Survey 2015-2016. Fairfax, United States of America: ICF International, 2016. | 218593 | 3510 | 3510 |
| United Republic of Tanzania | 2017 | Tanzania HIV Impact Survey 2016-2017 | Survey microdata | Admin 1 | Centers for Disease Control and Prevention (CDC), ICAP, Columbia University Mailman School of Public Health, Ministry of Health (Zanzibar), Ministry of Health, Community Development, Gender, Elderly and Children (MoHCDEC) (Tanzania), National AIDS Control Program (NACP) (Tanzania), National Bureau of Statistics (Tanzania), National Health Laboratory Quality Assurance and Training Centre (NHLQATC) (Tanzania), Office of the Chief Government Statistician (OCGS) (Zanzibar), Tanzania Commission for AIDS (TACAIDS), Zanzibar AIDS Commission (ZAC), Zanzibar Integrated HIV, Tuberculosis and Leprosy Program (ZIHTLP). Tanzania HIV Impact Survey 2016-2017. | 327591 | 12078 | 10883 |
| Zambia | 2000 | Zambia Sexual Behavior Survey 2000 | Survey microdata | Admin 2 | Central Board of Health (Zambia), Central Statistical Office (Zambia), MEASURE Evaluation Project, Carolina Population Center, University of North Carolina, University of Zambia, Zambian National AIDS Council. Zambia Sexual Behavior Survey 2000. Lusaka, Zambia: Central Statistical Office (Zambia). | 27924 | 1369 | 1369 |
| Zambia | 2003 | Zambia Sexual Behavior Survey 2003 | Survey microdata | Admin 2 | Central Board of Health (Zambia), Central Statistical Office (Zambia), MEASURE Evaluation Project, Carolina Population Center, University of North Carolina, Zambian National AIDS Council. Zambia Sexual Behavior Survey 2003. Lusaka, Zambia: Central Statistical Office (Zambia). | 27952 | 1990 | 1990 |
| Zambia | 2005 | Zambia Sexual Behavior Survey 2005 | Survey microdata | Admin 2 | Central Statistical Office (Zambia), Department for International Development (DFiD) (United Kingdom), Family Health International, MEASURE Evaluation Project, Carolina Population Center, University of North Carolina, Ministry of Health (Zambia), University of Zambia, Zambian National AIDS Council. Zambia Sexual Behavior Survey 2005. Lusaka, Zambia: Central Statistical Office (Zambia). | 27987 | 1900 | 1900 |
| Zambia | 2007 | Zambia Demographic and Health Survey 2007 | Survey microdata | GPS | Central Statistical Office (Zambia), Macro International, Inc. Zambia Demographic and Health Survey 2007. Fairfax, United States of America: ICF International. | 21117 | 6000 | 6000 |
| Zambia | 2009 | Zambia Sexual Behavior Survey 2009 | Survey microdata | Admin 2 | Central Statistical Office (Zambia), MEASURE Evaluation Project, Carolina Population Center, University of North Carolina, Ministry of Health (Zambia), University of Zambia, Zambian National AIDS Council. Zambia Sexual Behavior Survey 2009. | 59339 | 1902 | 1902 |
| Zambia | 2013-14 | Zambia Demographic and Health Survey 2013-2014 | Survey microdata | GPS | Central Statistical Office (Zambia), ICF International, Ministry of Health (Zambia), Tropical Diseases Research Centre, University Teaching Hospital (Zambia), University of Zambia. Zambia Demographic and Health Survey 2013-2014. Fairfax, United States of America: ICF International. | 77516 | 13478 | 13478 |
| Zambia | 2016 | Zambia Population-based HIV Impact Assessment 2016 | Survey microdata | Admin 2 | Centers for Disease Control and Prevention (CDC), ICAP, Columbia University Mailman School of Public Health, Ministry of Health (Zambia), Statistical Center for HIV/AIDS Research and Prevention (SCHARP), Tropical Diseases Research Centre, University of Zambia, Westat. Zambia Population-Based HIV Impact Assessment 2016. New York, New York: ICAP, Columbia University Mailman School of Public Health. | 287630 | 8389 | 7676 |
| Zimbabwe | 2005 | Zimbabwe - Chimanimani Behavioral Risks and HIV Serostatus Survey 2005 | Survey report | Admin 2 | Biomedical Research and Training Institute (Zimbabwe), Human Sciences Research Council, National Institute of Health Research, Ministry of Health and Child Welfare (NIHR) (Zimbabwe), Zimbabwe Central Statistics Office. Zimbabwe - Chimanimani Behavioral Risks and HIV Serostatus Survey 2005. | 333662 | 322 | 11 |
| Zimbabwe | 2005-06 | Zimbabwe Demographic and Health Survey 2005-2006 | Survey microdata | GPS | Central Statistical Office (Zimbabwe), Macro International, Inc. Zimbabwe Demographic and Health Survey 2005-2006. Fairfax, United States of America: ICF International. | 21163 | 6784 | 6784 |
| Zimbabwe | 2010-11 | Zimbabwe Demographic and Health Survey 2010-2011 | Survey microdata | GPS | ICF Macro, Zimbabwe National Statistics Agency. Zimbabwe Demographic and Health Survey 2010-2011. Calverton, United States of America: ICF Macro, 2012. | 55992 | 6788 | 6788 |
| Zimbabwe | 2014 | Zimbabwe Multiple Indicator Cluster Survey 2014 | Survey microdata | Admin 1 | United Nations Children's Fund (UNICEF), Zimbabwe National Statistics Agency. Zimbabwe Multiple Indicator Cluster Survey 2014. New York, United States of America: United Nations Children's Fund (UNICEF), 2015. | 152720 | 7561 | 6882 |
| Zimbabwe | 2015 | Zimbabwe Demographic and Health Survey 2015 | Survey microdata | GPS | ICF International, National Microbiology Reference Laboratory, Harare Central Hospital (NMRL) (Zimbabwe), Zimbabwe National Statistics Agency. Zimbabwe Demographic and Health Survey 2015. Fairfax, United States of America: ICF International, 2016. | 157066 | 8008 | 8008 |
| Zimbabwe | 2016 | Zimbabwe Population-based HIV Impact Assessment 2015-2016 | Survey report | Admin 1 | Ministry of Health, Zambia. Zambia Population-based HIV Impact Assessment (ZAMPHIA) 2016: Final Report. Lusaka, Ministry of Health. February 2019. http://phia.icap.columbia.edu | 287631 | 9271 | 2536 |

## Supplementary Table 2: Surveys excluded from this analysis.

*NID = Data source unique identifier in the Global Health Data Exchange.^1^ Additional information about each data sources is available *via* the GHDx, including information about the data provider and links to where the data can be accessed or requested (where available). NIDs can be entered in the search bar to retrieve the record for a particular source.

| **Country** | **Year(s)** | **Name** | **Citation** | **NID*** | **Rationale** |
| --- | --- | --- | --- | --- | --- |
| Nigeria | 2007 and 2012 | National HIV/AIDS and Reproductive Health Survey | Federal Ministry of Health (Nigeria), National Bureau of Statistics (Nigeria), National Population Commission (NPC), Society for Family Health (Nigeria), University College Hospital, Ibadan. Nigeria National HIV/AIDS and Reproductive Health Surveys 2007 and 2012. | 325046, 324443 | Estimates for MC were implausibly lower than estimates from independent survey series (2008 DHS, 2013 DHS, and 2016–2017 MICS). |
| Nigeria | 2008–10 | Living Standards Survey | National Bureau of Statistics (Nigeria). Nigeria Living Standards Survey 2008–2010. Abuja, Nigeria: National Bureau of Statistics (Nigeria). | 151719 | Estimates for MC were implausibly lower than estimates from independent survey series (2008 DHS, 2013 DHS, and 2016–2017 MICS). |
| São Tomé and Príncipe | 2014 | Sao Tome and Principe Multiple Indicator Cluster Survey 2014 | Global Fund to Fight Aids Tuberculosis and Malaria (GFATM), ICF International, National Center for Endemic Diseases (CNE) (Sao Tome and Principe), National Institute of Statistics (Sao Tome and Principe), United Nations Children's Fund (UNICEF), United Nations Development Programme (UNDP). Sao Tome and Principe Multiple Indicator Cluster Survey 2014. New York, United States: United Nations Children's Fund (UNICEF), 2016. | 214640 | National MC estimates reflect substantial differences from the rest of Western sub-Saharan Africa, which would break our model’s assumption to borrow information on MC coverage across space and time. |

## Supplementary Table 3: Data requiring age cross-walk.

| **Country** | **Year(s)** | **Name** | **Age Group** |
| --- | --- | --- | --- |
| Eswatini | 2017 | Lesotho Population-based HIV Impact Assessment 2016-2017 | 15-59 |
| Namibia | 2017 | Namibia Population-based HIV Impact Assessment 2017 | 15-64 |
| Uganda | 2016 | Uganda Population-based HIV Impact Assessment 2016-2017 | 15-64 |
| Zimbabwe | 2005 | Zimbabwe - Chimanimani Behavioral Risks and HIV Serostatus Survey 2005 | 15-24 |
| Zimbabwe | 2016 | Zimbabwe Population-based HIV Impact Assessment 2015-2016 | 15-64 |

## Supplementary Table 4: Number and designated name for first and second administrative level units

| Country | Names of first administrative level units | Total number of first administrative level units | Names of second administrative level units | Total number of second administrative level units |
| --- | --- | --- | --- | --- |
| Angola | Provinces | 18 | Municipalities | 163 |
| Benin | Departments | 12 | Communes | 77 |
| Botswana | Districts | 16 | Sub-districts | 30 |
| Burkina Faso | Regions | 13 | Provinces | 45 |
| Burundi | Provinces | 17 | Municipalities | 129 |
| Cameroon | Regions | 10 | Departments | 58 |
| Central African Republic | Prefectures | 17 | Sub-prefectures | 71 |
| Chad | Regions | 23 | Departments | 53 |
| Comoros | Autonomous Islands | 3 | Autonomous Islands | 3 |
| Côte d'Ivoire | Districts | 14 | Territories | 33 |
| Democratic Republic of the Congo | Provinces | 26 | Territories | 239 |
| Eritrea | Regions | 6 | Subregions | 58 |
| Eswatini | Regions | 4 | Tinkhundla | 55 |
| Ethiopia | Regions | 11 | Zones | 78 |
| Gabon | Provinces | 9 | Departments | 48 |
| Ghana | Regions | 10 | Districts | 216 |
| Guinea | Regions | 8 | Prefectures | 34 |
| Guinea-Bissau | Regions | 9 | Sectors | 37 |
| Kenya | Counties | 47 | Sub-counties | 290 |
| Lesotho | Districts | 10 | Constituencies | 10 |
| Liberia | Counties | 15 | Districts | 65 |
| Madagascar | Provinces | 6 | Regions | 22 |
| Malawi | Districts | 3 | Constituencies | 28 |
| Mali | Regions | 9 | Cercles | 50 |
| Mozambique | Provinces | 11 | Districts | 129 |
| Namibia | Regions | 13 | Constituencies | 107 |
| Niger | Regions | 8 | Departments | 64 |
| Nigeria | States | 37 | Local Government Areas | 769 |
| Republic of Congo | Departments | 12 | Districts | 89 |
| Rwanda | Provinces | 5 | Districts | 30 |
| Senegal | Regions | 14 | Departments | 45 |
| Sierra Leone | Provinces | 4 | Districts | 14 |
| South Africa | Provinces | 9 | Districts | 52 |
| Togo | Regions | 5 | Prefectures | 30 |
| Uganda | Districts | 128 | Counties | 200 |
| United Republic of Tanzania | Regions | 30 | Districts | 169 |
| Zambia | Provinces | 10 | Districts | 103 |
| Zimbabwe | Provinces | 10 | Districts | 60 |

## Supplementary Table 5: Validation metrics.

| **Level** | | **Mean error (percentage-points)** | **RMSE (percentage-points)** | **95% prediction interval coverage (%)** |
| --- | --- | --- | --- | --- |
| Country | In-sample | 0.01 | 0.58 | 98.33 |
|  | Out-of-sample | 0.59 | 3.56 | 96.96 |
| First administrative unit level | In-sample | 0.01 | 2.67 | 98.33 |
|  | Out-of-sample | 0.59 | 6.12 | 96.96 |
| Second administrative unit level | In-sample | 0.01 | 5.30 | 98.33 |
|  | Out-of-sample | 0.59 | 8.65 | 96.96 |

## Supplementary Table 6: Prior sensitivity analysis results.

| **Model results compared** | **Correlation** | | | **Mean absolute difference (PPT)** | | | **90^th^ percentile absolute difference (PPT)** | | | **Percent of overlapping uncertainty intervals** | | |
| --- | --- | --- | --- | --- | --- | --- | --- | --- | --- | --- | --- | --- |
|  | **Grid cell** | **Admin 1** | **Admin2** | **Grid cell** | **Admin 1** | **Admin 2** | **Grid cell** | **Admin 1** | **Admin 2** | **Grid cell** | **Admin 1** | **Admin 2** |
| Model 1 | 1 | 1 | 1 | 0.425 | 0.412 | 0.392 | 1.015 | 1.038 | 1.046 | 100 | 100 | 100 |
| Model 2 | 1 | 1 | 1 | 0.447 | 0.411 | 0.370 | 1.070 | 1.007 | 0.973 | 99.58 | 100 | 100 |
| Model 3 | 1 | 1 | 1 | 0.446 | 0.420 | 0.380 | 1.076 | 1.077 | 1.015 | 100 | 100 | 100 |
| Model 4 | 1 | 1 | 1 | 0.416 | 0.380 | 0.350 | 1.024 | 0.948 | 0.900 | 100 | 100 | 100 |
| Model 5 | 1 | 1 | 1 | 0.420 | 0.416 | 0.389 | 1.034 | 1.025 | 1.019 | 100 | 100 | 100 |

Supplementary Table 5 presents metrics of model comparison for the sensitivity analyses on hyper-prior selection. For each indicator, it presents a comparison between all given models described in appendix section 3 with the selected model, computing the correlation, mean absolute error, 90^th^ percentile of observed difference and percent of uncertainty interval overlap at the raster (5 × 5-km) level, first administrative level, and second administrative level.

## Supplementary Table 7: Fitted model parameters.

|  | **Central sub-Saharan Africa** | | | **Eastern sub-Saharan Africa** | | | **Southern sub-Saharan Africa** | | | **Western sub-Saharan Africa** | | |
| --- | --- | --- | --- | --- | --- | --- | --- | --- | --- | --- | --- | --- |
| **Parameter** | **0.025^th^ quantile** | **0.500^th^ quantile** | **0.975^th^ quantile** | **0.025^th^ quantile** | **0.500^th^ quantile** | **0.975^th^ quantile** | **0.025^th^ quantile** | **0.500^th^ quantile** | **0.975^th^ quantile** | **0.025^th^ quantile** | **0.500^th^ quantile** | **0.975^th^ quantile** |
| $\beta_{0}$ | 0.743 | 2.121 | 3.497 | 0.286 | 1.267 | 2.248 | -2.525 | -1.774 | -1.024 | 2.761 | 3.604 | 4.448 |
| $\beta_{1}$ | 1.148 | 3.272 | 5.398 | 0.363 | 1.12 | 1.876 | 0.878 | 1.464 | 2.05 | 0.437 | 1.495 | 2.551 |
| Standard Dev. for $\gamma_{c\left[ i \right]}$ | 0.0631 | 0.4463 | 2.3187 | 0.6426 | 1.1593 | 2.4693 | 0.4517 | 0.9006 | 2.0943 | 0.6313 | 1.1301 | 1.9209 |
| Range for $Z_{i,t}$ | 0.04 | 0.06 | 0.088 | 0.055 | 0.063 | 0.071 | 0.04 | 0.045 | 0.052 | 0.025 | 0.031 | 0.036 |
| Standard Dev. for $Z_{i,t}$ | 1.08 | 1.368 | 1.712 | 2.331 | 2.628 | 2.915 | 0.841 | 0.912 | 1.008 | 1.41 | 1.533 | 1.67 |
| ρ for $Z_{i,t}$ | 0.797 | 0.929 | 0.976 | 0.99 | 0.993 | 0.994 | 0.944 | 0.955 | 0.966 | 0.87 | 0.906 | 0.943 |
| Standard Dev. for $\epsilon_{i,t}$ | 0.1438 | 0.2984 | 0.5002 | 0.7464 | 0.7661 | 0.7806 | 0.3283 | 0.3611 | 0.4047 | 0.8798 | 0.9329 | 1.0185 |
| Supplementary Table 6 presents descriptive statistics for the posterior distribution of parameters in our geostatistical model (appendix section 3.1). As referenced above, $\beta_{0}$ is the intercept, $\beta_{1}$ is a fixed-effect on year, $\gamma_{c\left[ i \right]}$ is a country-level random effect for country *c* containing location *i,*$Z_{i,t}$ is a spatially and temporally correlated random effect for location ­­*i* and year *t,* and $\epsilon_{i,t}$ is an independent and identically distributed random effect for location ­­*i* and year *t.* | | | | | | | | | | | | |
